# Graphene-Skinned Fiber with Fine-Tunable Electrical Resistance via Radical and Substrate Engineering for Electromagnetic-Thermal Fabric

**DOI:** 10.1007/s40820-026-02117-8

**Published:** 2026-03-02

**Authors:** Jie Liang, Zhaochen Li, Fang Ye, Yuchen Cao, Yi An, Xiaomeng Fan, Qiang Song

**Affiliations:** Science and Technology On Thermostructural Composite Materials Laboratory, Northwestern Polytechnical University, Xi’an, 710072 People’s Republic of China

**Keywords:** Graphene, Fiber, Radical manipulation, Electromagnetic wave, Joule heating

## Abstract

This work demonstrates a radical-manipulation strategy for synthesizing graphene (Gr)-skinned SiO_2_ fabric via low-pressure chemical vapor deposition using methanol precursor. Controlled pyrolysis at high temperature regulated C_1_/C_2_/C_6_ radical ratios, enabling microstructure engineering. Substrate effects governed bilayer evolution. SiO_2_ imposed lower adsorption energy of C_1_ and higher diffusion barriers of radical compared to Gr, promoting edge defects in subsurface G1-type Gr layers, whereas reduced substrate constraints facilitated low-defect G2-type Gr growth on top of G1-type Gr. Synergistic control of gas-phase kinetics and substrate dynamics enabled fine-tunable sheet resistance (26–150 Ω sq^−1^), establishing Gr-skinned fibers as multifunctional platforms for integrated electromagnetic-thermal management systems. When addressing the needs of electromagnetic communication and electrothermal deicing, laser-etched band-pass frequency selective surface structures of Gr-skinned fabric were fabricated to achieve electromagnetic wave (EMW) transmittance while maintaining Joule heating capability. A sandwich structure was prepared by laminating the Gr-skinned fabric with EMW transparent sheets exhibiting voltage-dependent transmittance, simultaneously sustaining broadband transmission and effective heating. This work demonstrates a strategy to mitigate the longstanding conductivity-EMW transparency trade-off in Gr-functionalized fibers through a multiscale engineering that coordinates microscopic structural regulation with macroscopic patterning, thereby unlocking next-generation smart composites for 5G/6G wearables, aerospace radomes, and beyond.

**Supplementary Information:**

The online version contains supplementary material available at 10.1007/s40820-026-02117-8.

## Highlights

- The composition of C_1_/C_2_/C_6_ radicals is strategically regulated by temperature, dictating the growth of either defective or highly textured graphene microstructures.
- A synergistic strategy involving radical manipulation and substrate effect via methanol- chemical vapor deposition enables precise control of graphene microstructure, allowing fine tuning of sheet resistance from 26 to 150 Ω sq^−1^.
- Laser-patterned band-pass frequency selective surface and a sandwich structure synergistically achieve excellent broadband wave transmission (7.29 GHz) and effective Joule heating (72 °C).

**Supplementary Information:**

The online version contains supplementary material available at 10.1007/s40820-026-02117-8.

## Graphical abstract

**Figure:**
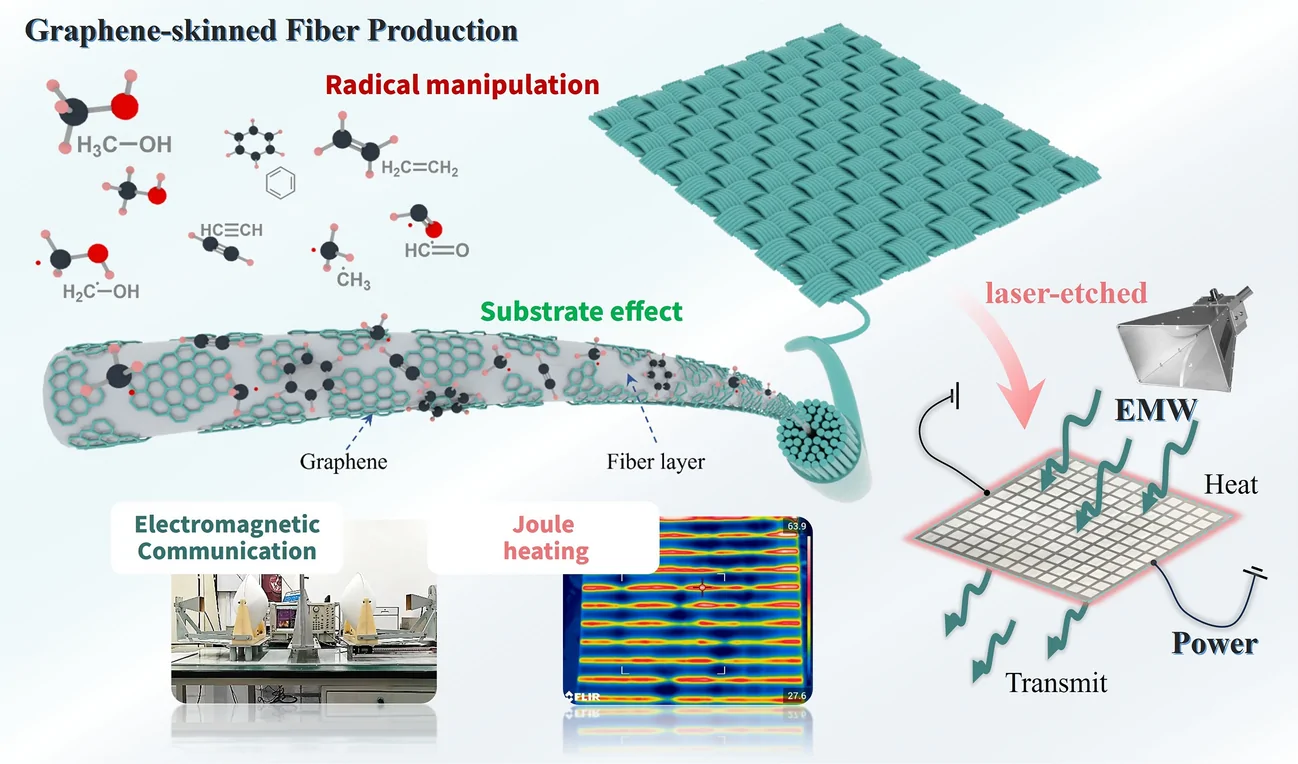

## Introduction

Owing to their exceptional intrinsic mechanical strength, fibrous materials are widely employed in fabricating fiber-reinforced composites (FRCs) with metallic, ceramic, or polymeric matrices. The rapid advancement of electronic information technologies and living standards has generated new demands for high load-capacity FRCs with multifunctional capabilities, including Joule heating, electromagnetic wave (EMW) communication, absorption, shielding, and deicing [1–3]. Conventional fibers (e.g., glass, carbon, aramid) are typically categorized as either high-resistance or high-conductivity materials, rendering them inadequate for such multifunctional requirements. Consequently, fiber modification has become essential to address contemporary application needs. Graphene (Gr), a two-dimensional atomic crystal, exhibits numerous superior physical properties [4–6]. Thus, the electrical properties can be improved by producing graphene-modified fibers (termed Gr-skinned fiber) [7, 8]. Nevertheless, insufficient electrical tunability of current Gr-skinned fiber restricts their applicability across diverse scenarios.

Electrical property tuning refers to the strategic modulation of electrical conductivity within a target range through process control or other methodologies. Liu et al. employed a wet-chemistry assembly process to modulate the electrical properties of aramid fibers by adsorbing varying amounts of Gr and carbon nanotubes onto their surface. However, as their approach did not involve engineering the Gr microstructure, it had a limited ability to achieve precise control over the electrical characteristics [9]. Park et al. utilized dopants including melamine, red phosphorus, and sulfur powder to synthesize N-doped, P-doped, and S-doped reduced graphene oxide (rGO) fiber with elemental concentrations of 6.52, 4.43, and 2.06 at%, respectively. The P-doped and S-doped rGO fiber exhibited conductivity enhancements of 33% and 28% over pristine rGO fiber, while N-doped rGO fiber achieved the highest conductivity of 1.11 × 10^4^ S m^−1^ [10]. Complementarily, Witjaksono et al. demonstrated N-doped Gr with a maximum nitrogen concentration of ~ 5.51 at%, confirming monotonic conductivity improvement with increasing doping levels [11]. Spectroscopic analysis revealed a pyrrolic-N-dominated configuration in N-rGO samples, which facilitates π-bond reinforcement in carbon lattices by suppressing Stone–Wales defects and establishing electron percolation pathways. This mechanism effectively eliminates conduction gaps and enhances n-type conductivity. Nevertheless, doping strategies face inherent challenges in precise control and fine-tunable limitations due to dopant inhomogeneity and stability issues.

During chemical vapor deposition (CVD), high-temperature pyrolysis of carbon precursors generates alkane-derived molecular fragments, which serve as critical gaseous intermediates for subsequent Gr deposition [8, 12]. Modulating these radical reactions represents a primary pathway for controlling intermediate concentrations and, consequently, tailoring Gr microstructure. C_1_ (CH_3_·), C_2_ (C_2_H_2_), and C_6_ (C_6_H_6_) species constitute key deposition precursors. C_1_/C_2_ fragments promote isotropic or low-texture pyrolytic carbon formation while providing high-reactivity carbon sources to sustain deposition rates. C_6_ aromatics facilitate stacking into highly ordered lamellar structures due to their spatial configuration [13–15]. Strategic balancing of these precursor ratios enables controlled transitions from isotropic to highly textured Gr architectures. When utilizing oxygen-containing precursors like methanol (CH_3_OH), pyrolysis co-generates H_2_O and CO_2_ byproducts that induce edge etching effects on carbon clusters [16, 17]. Precursor concentration modulation thus provides a viable route for Gr manipulation crystallographic orientation and electrical properties.

In this work, Gr was synthesized on SiO_2_ fiber surfaces via low-pressure chemical vapor deposition (LPCVD) using CH_3_OH as the carbon precursor. Through elementary reaction modulation, controlled ratios of C_1_, C_2_, and C_6_ species were achieved. Under lower temperatures, incomplete CH₃OH pyrolysis yielded C_1_-dominant precursors, promoting multi-site nucleation on rough fiber surfaces. This inhibited sufficient crystallite growth, resulting in low-texture Gr with reduced electrical conductivity. Conversely, higher temperatures facilitated complete pyrolysis, enriching C_6_ species that suppressed nucleation density and enabled large-crystallite, high-texture Gr with enhanced conductivity. H_2_O and CO_2_ byproducts induced edge etching, preferentially facilitating multilayer Gr growth. As deposition progressed, substrate effects diminished significantly, producing stratified Gr layers with distinct structural properties between basal and surface regions. Consequently, synergistic precursor control and substrate effect modulation enabled fine-tuning of sheet resistance within 26–150 Ω sq^−1^. To address multifunctional electro-thermal requirements, FSS-patterned Gr-skinned SiO_2_ fabric was fabricated via galvanometer laser scanning. The architecture exhibited a remarkable electro-thermal synergetic performance, with a surface temperature of 80 °C being attained when connected to an external 105 V voltage. Concurrently, 70% EMW transmittance was maintained across a frequency range of 14.7–18 GHz. This work establishes a methodology spanning atomic-scale structural control to macroscale functional design, highlighting Gr-skinned fiber as promising candidate materials for multifunctional composite systems.

## Experimental Section

### Preparation of Gr-Skinned SiO_2_ Fabric

Gr was deposited on SiO_2_ fiber fabric using CH_3_OH as the carbon precursor to synthesize Gr-skinned fabric. The substrate was positioned within a custom-built deposition furnace, followed by vacuum evacuation and temperature ramping. Upon reaching the target deposition temperature (1000–1200 °C), methanol vapor was introduced under controlled pressure conditions (6–8 kPa). Following the prescribed deposition time (0.5–3 h), the gas supply was terminated and systematic cooling was initiated. All samples are designated in the nomenclature *x*–*y*, where *x* denotes the deposition temperature, and *y* represents the deposition time.

### Preparation of ET-Gr-Skinned SiO_2_ Fabric

The Gr-skinned fabric was positioned on a laser scanning stage, where the pre-designed etching pattern was imported into the control system to initiate laser ablation. Processing parameters were configured as follows: laser power = 40 W, pulse frequency = 100 Hz, scan velocity = 1000 mm s^−1^, line width = 0.1 mm, and line distance = 0.1 mm.

### Electromagnetic Parameters Testing

Reflectance (R), transmittance (T), and absorptivity (A) were measured via the waveguide method, employing a vector network analyzer (MS4644A, Anritsu, Japan) within the 8.2–12.4 GHz. Specimen size of Gr-skinned fabric is 22.86 × 10.16 × 0.25 mm^3^. Furthermore, large-scale samples of SiO_2_ fabric, Gr-skinned fabric, and etched (ET) Gr-skinned fabric were characterized using the free-space method across the 8–18 GHz frequency range, and the size of specimens is 180 × 180 × 0.25 mm^3^. A comprehensive evaluation of the electromagnetic and Joule heating properties of an integrated sandwich structure composed of an ET Gr-skinned fabric sandwiched between two acrylic plates was conducted. The structure consists of a 3-mm thick acrylic top layer, a fabric interlayer, and a 3-mm thick acrylic bottom layer. After fabricating the composite sandwich structure, it was connected to a power supply and placed in a free-space test setup, allowing for the simultaneous measurement of its electromagnetic performance during the Joule heating process.

### Characterization

The morphology and microstructure of the samples were characterized by scanning electron microscope (SEM, Helios G4, FEI, USA), transmission electron microscope (TEM, Themis Z, FEI, USA), X-ray photoelectron spectroscopy (XPS, Axis Supra, Kratos, UK), and Raman spectroscopy (InVia, Renishaw, excited by a 532 nm He–Ne laser with a laser spot size of 1 µm^2^). To facilitate TEM observation of Gr structures, the Gr-skinned SiO_2_ fibers were immersed in 0.1 mol L^−1^ HF for 4 h, followed by ethanol washing to obtain pure Gr samples. The carbon content of the samples was quantified using a carbon–sulfur analyzer (LECO, CS844, USA). For the measurement of sheet resistance (*R*_s_), a four-probe test was conducted using an M-3 Mini type four-probe tester (Suzhou Ijingge Electronic Co., Ltd., China) with a probe spacing of 2 mm, and measurements were performed at 10 mm intervals across the sample surface.

All calculations were carried out for the material in the framework of density functional theory (DFT) using the Vienna Ab initio Simulation Package (VASP 6.3.0). The generalized gradient approximation (GGA) of the Perdew–Burke–Ernzerhof (PBE) function was used to describe the exchange–correlation energy. The projected augmented wave (PAW) method and pseudopotentials were used to describe the interactions between valence electrons and ions. To ensure the efficiency of the computational results and parallel computing. A 4 × 4 × 1 k-point grid under Monkhorst–Pack is used in the optimization process, and 500 eV truncation energy is set. The lattice parameters and ionic positions of all crystals were fully relaxed, and the convergence criteria for the total energy of all relaxed atoms and the final force were 10^−5^ and 0.05 eV Å^−1^, respectively. The SiO_2_ fiber surface was assumed to have an α-SiO_2_ (001) structure. It should be noted that our calculations employ an idealized flat SiO_2_ (001) surface to reveal the fundamental influence of its polarized chemical nature on precursor behavior. The roughness and curvature of actual SiO_2_ fibers, by introducing microscopic defects, would further increase the effective diffusion barriers and provide additional nucleation sites. Consequently, the restrictive effect of the SiO_2_ substrate in real environments is likely more pronounced than our model predicts.

## Results and Discussion

### Radical Manipulation

**Fig. 1:**
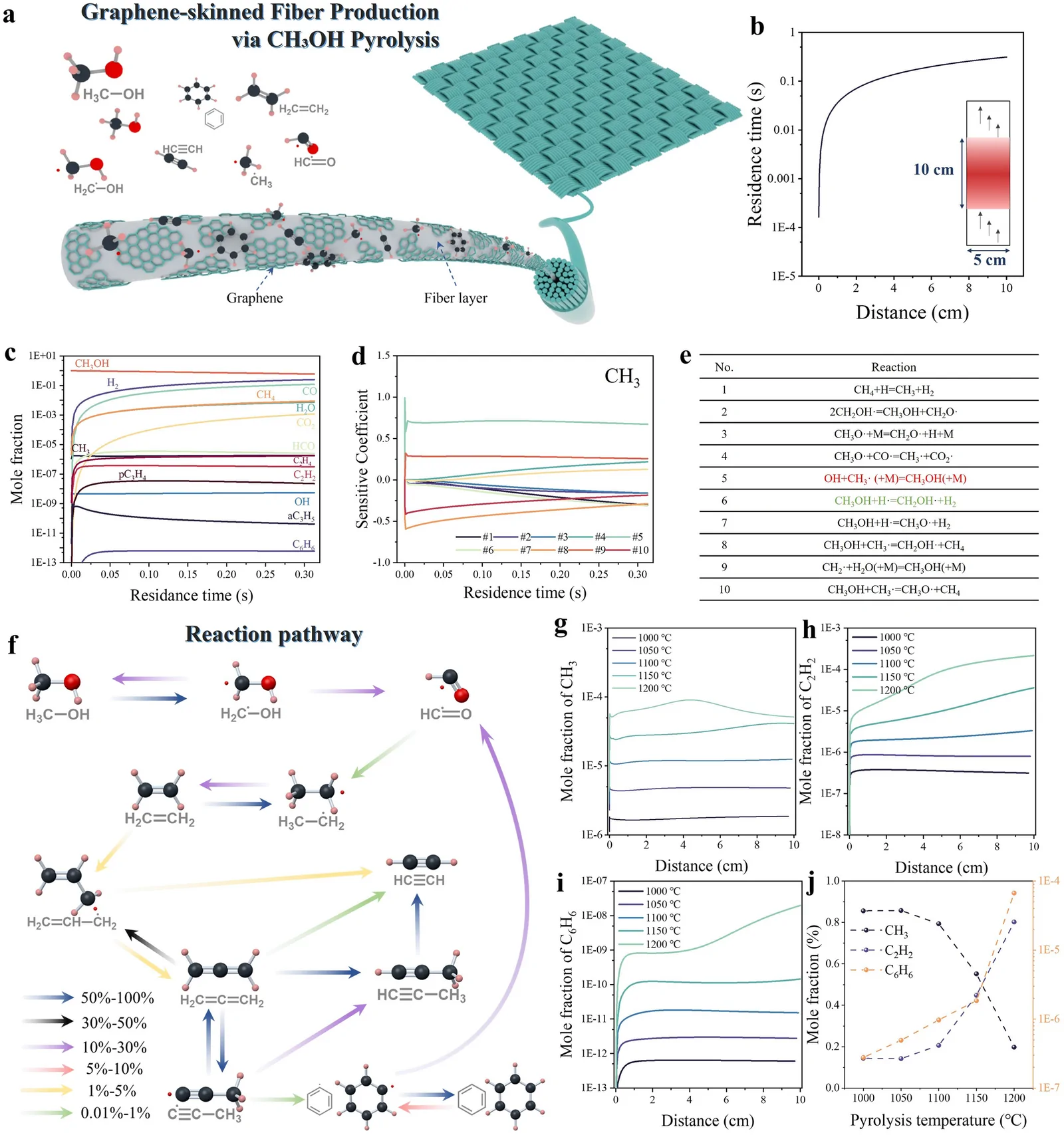
**a** Schematic diagram of CH_3_OH high-temperature pyrolysis. **b** Relaxation time at different positions in the reactor (inset: reactor model), **c** variation of different gas molar fractions with residence time, **d** sensitivity analysis of CH_3_·, **e** the top 10 elementary reactions affecting the sensitivity of the CH_3_·, **f** reaction pathway at a relaxation time of 0.15 s, **g** molar fraction of CH_3_·, **h** molar fraction of C_2_H_2_, **i** molar fraction of C_6_H_6_ at different temperatures, **j** ratio of the three gases (CH_3_·, C_2_H_2_, and C_6_H_6_)

The gas-phase reaction process provides the essential material foundation for solid-phase deposition and represents the dominant reaction pathway in gas pyrolysis (Fig. 1a). To accurately describe methane gas-phase pyrolysis within CVD and quantify concentrations of pyrolysis products, elucidating the underlying gas-phase reaction mechanism of methane pyrolysis is imperative. CVD processes typically employ tubular reactors, commonly approximated as plug-flow reactors [18]. CH_3_OH gas is introduced into such a plug-flow reactor to analyze its gas-phase pyrolysis behavior. The reaction zone consists of a straight cylindrical tube devoid of internal structures, thus resulting in a low specific surface area. Consequently, the deposition rate of solid pyrolytic carbon approaches zero, rendering it negligible. CH_3_OH is vaporized via a water bath and fed into the reactor inlet, where reactions proceed at 1273 K under a pressure of 8 kPa. Within the reaction zone (10 cm of length, 5 cm of diameter, Fig. 1b), gases react during a defined residence time before products exit through the outlet. Figure 1c illustrates residence times at distinct axial positions, indicating a progressive increase toward the reactor outlet. Figure 1d depicts species mole fractions, revealing that CO_2_ and H_2_O, byproducts of CH_3_OH significantly exceed the concentrations of hydrocarbons such as CH_3_·, C_2_H_2_, and C_6_H_6_.

In the CVD of carbon-based materials, Gr deposition reactions are primarily classified into three categories (C_1_, C_2_, and C_6_) based on gaseous carbon source species capable of deposition. Prior mechanistic studies indicate that methyl radicals (CH_3_·) serve as the dominant carbon source for Gr deposition. Acetylene (C_2_H_2_) deposits Gr via surface addition reactions, while increased concentrations of C_6_ species (e.g., benzene rings) enhance the structural ordering of the deposited pyrolytic carbon [19–21]. CO_2_ enables selective etching of small crystallites, thereby reducing grain boundary density and enhancing Gr texture [22]. Qi et al. demonstrated that H_2_O etches edges of upper-layer Gr while promoting basal-layer growth, facilitating uniform multilayer Gr formation [23]. Consequently, the relative concentrations of three carbon radicals and two byproducts critically govern Gr’s crystallographic structure and defect architecture.

Sensitivity analysis was employed to evaluate the influence of elementary reactions on the concentrations of major gaseous species during pyrolysis [24, 25]. The sensitivity coefficient for a given species is defined as the partial derivative of its mole fraction with respect to the kinetic parameters of the reaction. A positive sensitivity value indicates that the elementary reaction promotes the formation of the species, whereas a negative value signifies that it facilitates consumption. The absolute magnitude of the sensitivity reflects the strength of this promoting effect. Figure 1e displays the sensitivity coefficients affecting methane (CH_4_) concentration. The forward reaction OH· + CH_3_· (+ M) → CH_3_OH (+ M) exhibits the highest positive sensitivity, indicating its dominant role in enhancing CH_4_ formation. Conversely, the reaction CH_3_OH + H· → CH_2_OH· + H_2_ demonstrates negative sensitivity, confirming its inhibitory effect on CH_4_ generation. Figure S1 shows the sensitivity coefficients of the main reactions affecting C_2_H_2_ and C_6_H_6_.

To elucidate species transformation pathways during CH_3_OH gas-phase pyrolysis in CVD, dominant reaction networks were analyzed at a residence time of 0.3 s. Initial CH_3_OH consumption proceeds through two primary pathways: (1) thermal decomposition (CH_3_OH → CH_2_OH· + H·) and (2) radical-mediated reaction (CH_4_ + CH_3_· → C_2_H_5_· + H_2_). The hydroxymethyl radical (CH_2_OH·) subsequently reacts with formaldehyde (CH_2_O) to form formyl radical (HCO·) and CH_3_OH. As revealed in Fig. 1f, HCO· serves as the fundamental precursor for C_2_ and C_6_ species generation. However, ·HCO exhibits high reactivity and predominantly decomposes to H· and CO (HCO· → H· + CO), with less than 1% participating in ethyl radical formation via reaction with C_2_H_4_. This low HCO· to C_2_H_5_· conversion efficiency directly accounts for the minimal C_6_H_6_ mole fraction (~ 10^−12^).

To modulate key carbon source concentrations, CH_3_OH pyrolysis products were computationally evaluated across varying temperatures. Figures 1g–i and S2 demonstrate that elevated temperatures reduce residence time while concurrently increasing mole fractions of CH_3_·, C_2_H_2_, and C_6_H_6_. The ratio of C_2_H_2_ and C_6_H_6_ in the three gases gradually increases, while the ratio of CH_3_· decreases (Fig. 1j). Higher concentrations of gaseous products promote deposition reactions of C_2_ and C_6_ species, thus expanding pyrolytic carbon deposition pathways. These enable structural evolution toward either perfect hexagonal rings through single-precursor deposition or paired pentagon-heptagon topological defects via combined precursors. Consequently, deposition rates increase significantly, facilitating the growth of highly oriented Gr-like nanostructures [26–28]. The offline gas chromatographic analysis conducted under actual CVD conditions is presented in Fig. S3. Although quantitative deviations are observed due to sampling limitations and model idealization, the experimental data are shown to be in high qualitative agreement with the chemical kinetic simulations. An increase in deposition temperature is demonstrated to enhance the content of CH_4_ and C_2_H_2_. Consequently, the mechanism, whereby the Gr microstructure is modified through the regulation of key radical distribution by temperature, is robustly validated.

### Microstructure of Graphene

**Fig. 2:**
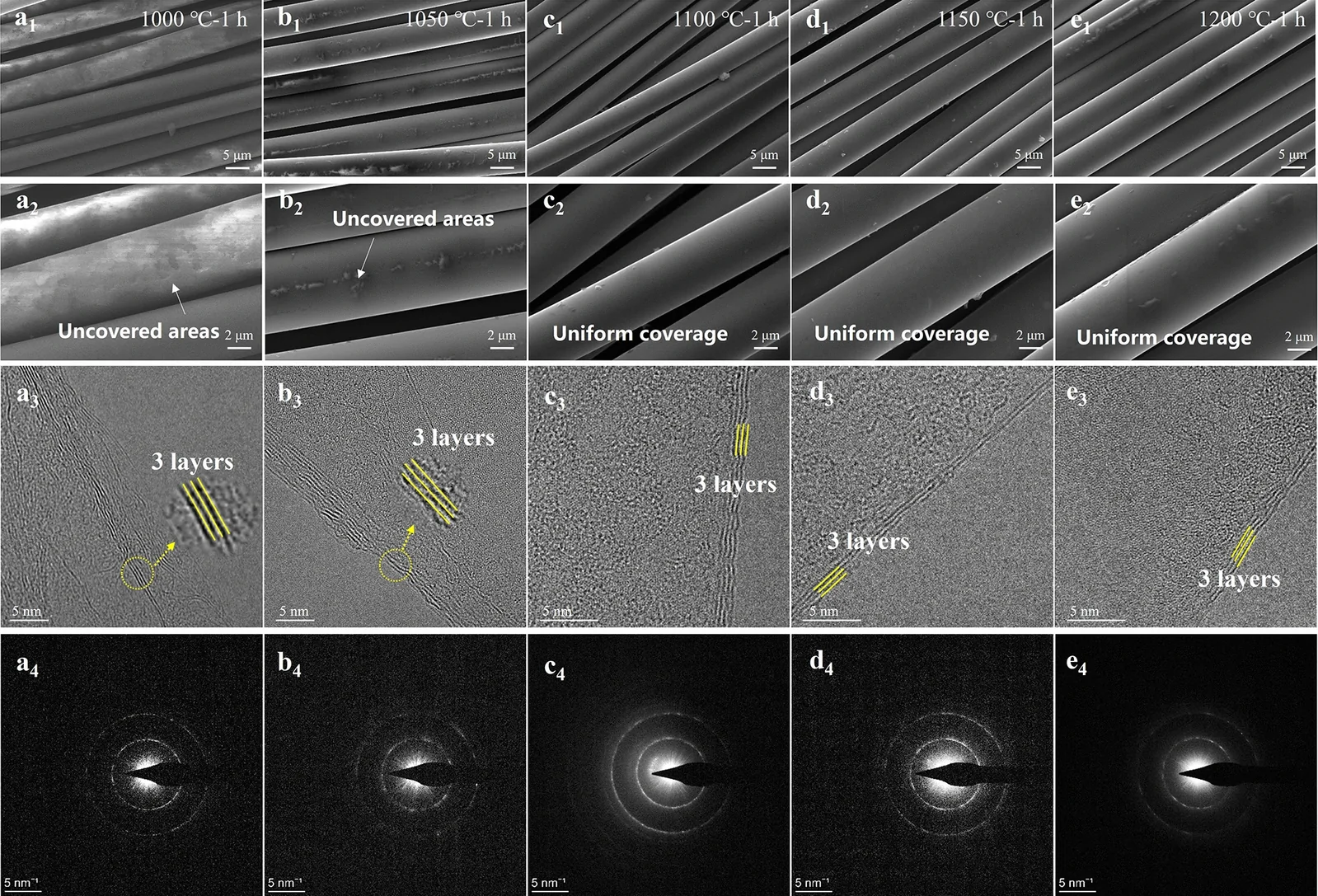
Micromorphology of Gr-skinned SiO_2_ fiber: **a**_**1**_**-a**_**2**_ 1000 °C-1 h, **b**_**1**_**-b**_**2**_ 1050 °C-1 h, **c**_**1**_**-c**_**2**_ 1100 °C-1 h, **d**_**1**_**-d**_**2**_ 1150 °C-1 h, **e**_**1**_**-e**_**2**_ 1200 °C-1 h. Microstructure of Gr **a**_**3**_ 1000 °C-1 h, **b**_**3**_ 1050 °C-1 h, **c**_**3**_ 1100 °C-1 h, **d**_**3**_ 1150 °C-1 h, **e**_**3**_ 1200 °C-1 h. Selective electron diffraction of Gr: **a**_**4**_ 1000 °C-1 h, **b**_**4**_ 1050 °C-1 h, **c**_**4**_ 1100 °C-1 h, **d**_**4**_ 1150 °C-1 h, **e**_**4**_ 1200 °C-1 h

Building on the CH_3_OH pyrolysis calculations, temperature significantly influences the types and concentrations of radical species generated during pyrolysis, thereby governing the structural characteristics of deposited Gr. Consequently, we experimentally investigated the impact of deposition temperature on Gr structure. Using CH_3_OH as the carbon source with a fixed deposition duration of 1 h, Gr-skinned SiO_2_ fiber were synthesized at five discrete deposition temperatures (1000, 1050, 1100, 1150, and 1200 °C), designated as 1000-1, 1050-1, 1100-1, 1150-1, and 1200-1, respectively. Figures 2a_1_–e_1_ and a_2_–e_2_) present SEM images of the five specimens, revealing incomplete Gr coverage on fiber surfaces for 1000–1 and 1050-1, indicating insufficient surface deposition under lower temperatures. Uniform Gr deposition was achieved at temperatures exceeding 1050 °C. Carbon content (mass fraction) quantified via carbon–sulfur analysis demonstrates minimal fluctuations across triplicate measurements, with values increasing proportionally to deposition temperature (Fig. S4). The average carbon contents for the five specimens were 0.048%, 0.054%, 0.065%, 0.080%, and 0.097%, respectively. Consistent with CH_3_OH pyrolysis simulations, deposition uniformity correlates directly with deposition kinetics. Elevated temperatures enhance CH_3_OH decomposition efficiency, yielding higher deposition rates, which aligns precisely with computational pyrolysis results.

Microstructural analysis of Gr was performed via TEM, as shown in Fig. 2a_3_–e_3_. No significant variation in Gr thickness was observed across temperatures, with all specimens maintaining approximately 3 layers. However, distinct textural evolution emerged with increasing temperature. Selected-area electron diffraction (SAED) patterns confirm polycrystalline structures for all specimens (Fig. 2a_4_–e_4_). The 1050-1 sample displays sparser diffraction spots within the rings, indicating larger crystalline domains and fewer grain boundaries (edge defects). In contrast, 1100-1 exhibits substantially increased spot density with bright, continuous rings, signifying heightened boundary defects. As temperature increases further, reduced spot density in SAED patterns demonstrates the population of differently oriented crystals diminished.

XPS analysis of the C 1*s* bonding states in Gr was performed (Fig. S5). Peak deconvolution reveals four distinct bonding configurations: C–C bonds (284.8 eV), defect-associated peaks (primarily C–H, 285.4 eV), C–O bonds (286.5 eV), and π-π transitions (291.0 eV) [29–31]. The C–C bonds correspond to *sp*^2^ hybridization, serving as an indicator of graphitic ordering. Defect peaks signify edge defects, while C–O bonds reflect oxygen-induced *sp*^3^ hybridization. Quantitative analysis across deposition temperatures (Fig. S5) demonstrates a non-monotonic temperature dependence: C–C content initially increases, declines at intermediate temperatures, and subsequently rises again, peaking at 1050 °C. Conversely, defect concentration follows an inverse trend, reaching its maximum at 1100 °C. These XPS results corroborate TEM observations, collectively indicating altered structural characteristics in Gr synthesized above 1050 °C.

**Fig. 3:**
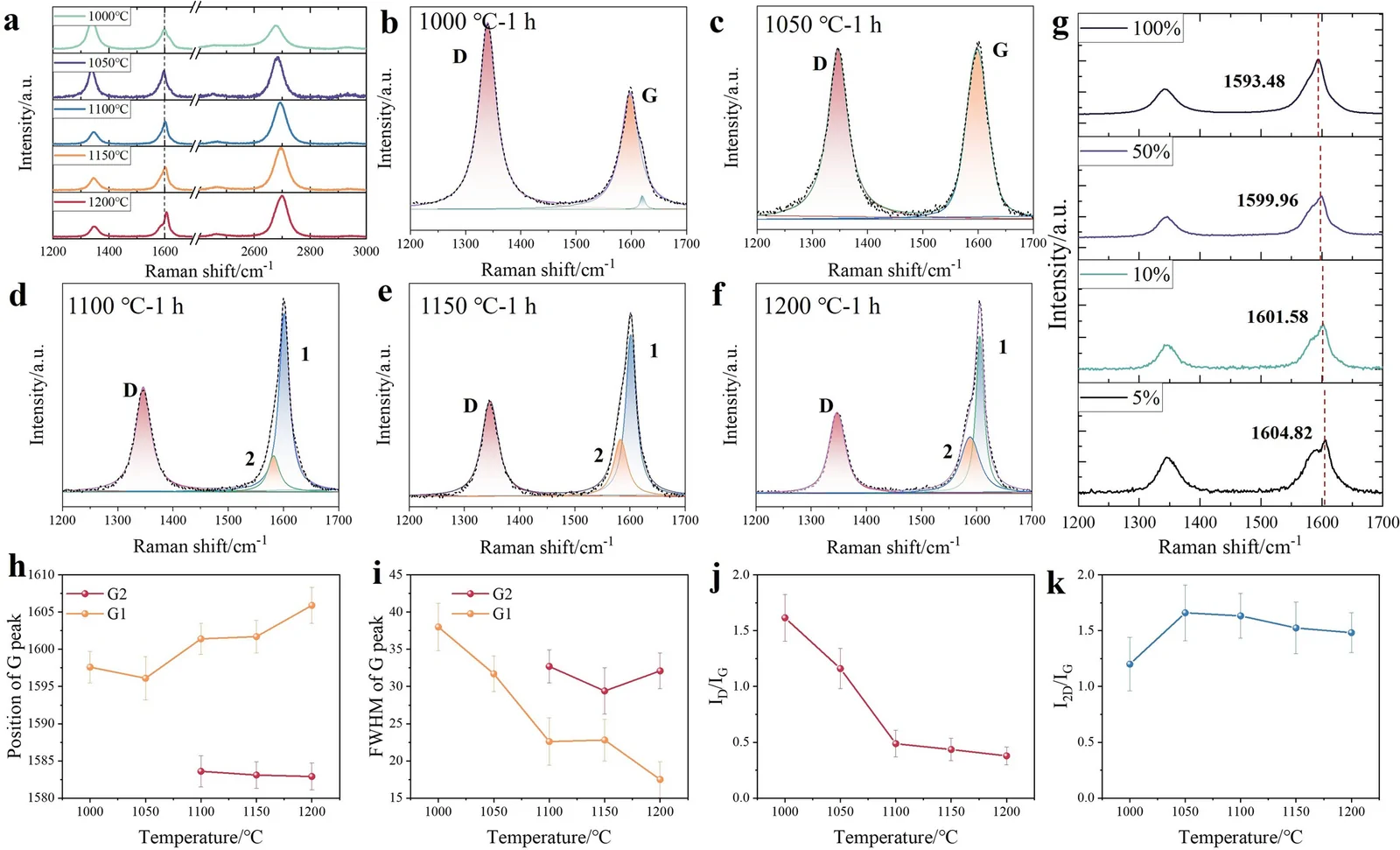
Raman spectra of Gr at different deposition temperatures: **a** Broadband spectrum, fine spectrum of **b** 1000 °C-1 h, **c** 1050 °C-1 h, **d** 1100 °C-1 h, **e** 1150 °C-1 h, **f** 1200 °C-1 h. **g** Raman spectra of Gr at different laser intensities. **h** G peak position, **i** FWHM of G peak, **j** I_D_/I_G_, **k** I_2D_/I_G_

Raman spectroscopy, being exquisitely sensitive to carbon structural features, was employed for further microstructural characterization of Gr, as presented in Figs. 3a and S6. The spectra exhibit characteristic D, G, and 2D peaks, confirming the Gr structure. The D band at 1350 cm^−1^ arises from lattice defects, predominantly armchair-edge defects. The G band at ~ 1580 cm^−1^ corresponds to in-plane vibrational modes of *sp*^2^-hybridized carbon atoms. Crucially, both the position and full width at half maximum (FWHM) of the G band serve as metrics for structural disorder manifested through distorted hexagonal rings and chains: Increased disorder typically leads to a blue shift while broadening the FWHM. The 2D band at ~ 2700 cm^−1^ indicates high-quality Gr and exhibits layer-dependent intensity, diminishing with increasing layer count. Additionally, the D′ band at ~ 1625 cm^−1^ is diagnostic of zigzag-edge defects [32–34]. Due to its spectral proximity to the G band, deconvolution was essential to resolve overlapping features (Fig. 3b–f). While the 1000-1 specimen shows distinct D, G, and D′ bands, the 1050-1 sample displays D′ band disappearance and reduced D-band intensity. Notably, above 1050 °C, the G-band region resolves into two overlapping peaks. A low-intensity peak at lower wavenumbers (Peak 2) and a high-intensity peak at higher wavenumbers (Peak 1).

According to Cançado et al. [35], when probing bilayer carbon materials with distinct structural features, laser-intensity-dependent G-band shifts occur due to thermal effects. Guided by this finding, Raman analysis was performed on Gr at laser intensities of 5%, 10%, 50%, and 100% (Fig. 3g). Increasing laser intensity induces progressive red shifting of Peak 1, while Peak 2 remains spectrally invariant (Fig. 3h–k). This divergence likely originates from differential thermal dissipation pathways: Surface Gr layers experience efficient heat dissipation, whereas underlying Gr undergoes thermal expansion due to constrained heat transfer through the thermally insulating fiber substrate. Consequently, Peak 1 and Peak 2 likely originate from subsurface and surface Gr layers, respectively. Furthermore, the relative intensities of G1 and G2 bands correlate with their growth chronology: The subsurface Gr layer nucleates preferentially, achieving higher deposition yield that manifests as enhanced G1 band intensity. Surface Gr initiates nucleation only after uniform subsurface growth, resulting in lower areal density due to shorter deposition time and thus diminished G2 intensity. Further analysis in conjunction with carbon content test results, specimens 1000-1 and 1050-1 exhibit minimal deposition yields and slow growth kinetics, explaining the absence of G2-type Gr. Conversely, accelerated deposition rates in 1100-1, 1150-1, and 1200-1 promote progressive formation of surface G2-type Gr with increasing temperature.

### Substrate Effect

**Fig. 4:**
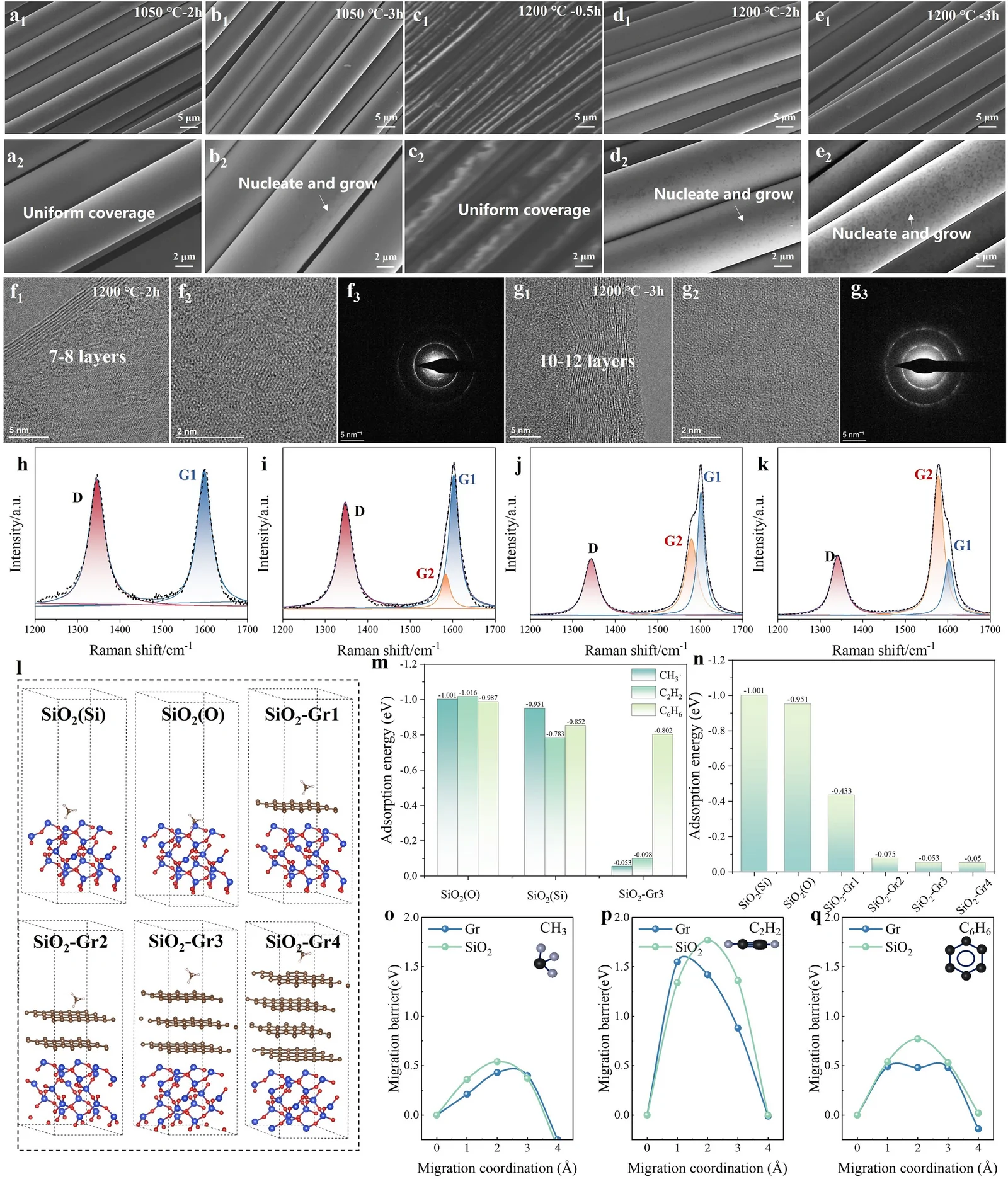
Micromorphology of Gr-skinned SiO_2_ fiber: **a** 1050 °C-2 h, **b** 1050 °C-3 h, **c** 1200 °C-0.5 h, **d** 1200 °C-2 h, **e** 1200 °C-3 h. Microstructure of Gr: **f**_**1**_**-f**_**2**_ 1200 °C-2 h, **g**_**1**_**-g**_**2**_ 1200 °C-3 h. Selective electron diffraction of Gr: **f**_**3**_ 1200 °C-2 h, **b**_**4**_ 1200 °C-3 h. Raman spectra of Gr at different deposition times: **h** 1050 °C-2 h, **i** 1050 °C-3 h, **j** 1200 °C-2 h, **k** 1200 °C-3 h. **l** Molecular model, **m** adsorption energy of CH_3_·, C_2_H_2_, and C_6_H_6_ on the surface of SiO_2_ and SiO_2_-Gr3, **n** adsorption energy of CH_3_· on the surface of SiO_2_, SiO_2_-Gr1, SiO_2_-Gr2, SiO_2_-Gr3 and SiO_2_-Gr4. Migration energy barriers of **o** CH_3_·, **p** C_2_H_2_, **q** C_6_H_6_ on two different substrates

To further validate the bilayer Gr architecture, deposition time was extended. Figures 4a1–e1 and a2–e2 present SEM images of specimens synthesized at 1050 and 1200 °C across different deposition times. At 1050 °C with inherently slower deposition rate, complete Gr coverage on SiO_2_ fiber requires deposition time more than 2 h (Fig. 4a_2_). At 1200 °C, discontinuous Gr coverage persists during initial deposition less than 1 h. Prolonged deposition achieves uniform surface coverage, with discernible proliferation and dimensional growth of densely packed nanosheets (multilayer island, Fig. 4e_2_). The Gr content at different deposition times at 1200 °C was tested using a carbon and sulfur analyzer. The results showed that it increased with the deposition time, and the average values of carbon content of the 1200-0.5, 1200-1, 1200-2, and 1200-3 were 0.072%, 0.097%, 0.109%, and 0.132%, respectively. Corresponding TEM characterization (Fig. 4f_1_–g_1_) reveals layer multiplication from 3 to 10 layers with deposition time increase. Figure 4f_2_–g_2_ displays the superimposed in-plane projection of multi-layer graphene. The observed complexity in the carbon atom arrangement is due to the overlapping of several graphene lattices with different crystallographic orientations. SAED (Fig. 4f_3_–g_3_) further corroborates this stratification, showing increased spot density in diffraction patterns indicative of enhanced crystallite population with different orientations.

Raman results showed that the G2 peak appeared in the sample at 1050 °C when the deposition time reached 3 h. The deposition time was extended at 1200 °C, and the G2 peak gradually enhanced and exceeded the G1 peak. In order to provide a comprehensive explanation for the microstructure evolution of Gr with the change of deposition time, we thoroughly investigated the Raman spectral evolution presented in Fig. S7, which covers samples prepared from 1000 to 1200 °C for 1 to 3 h. As demonstrated in Fig. 4h, i, more comprehensively, in Fig. S7a, the Gr prepared at 1000 °C exhibited G2-type Gr after 3 h, which can be attributed to the low deposition rate at this temperature. The full spectral series in Fig. S7 shows that this resulted in uniform coverage of the substrate surface after 2 h. The I_D_/I_G_ of different deposition times was calculated to be 1.62, 1.25, and 0.86, respectively. The monotonically decreasing I_D_/I_G_ trend shown in Fig. S7 is due to the fact that the G1-type Gr is still in the growth stage before 2 h. As the growth progresses, the Gr sheet gradually grows and the edge defect content decreases. The I_2D_/I_G_ of different deposition times at 1000 °C is 0.91, 0.91, and 0.82, respectively. This finding indicates that the number of Gr layers remains constant when the deposition time is less than 2 h. These data from Fig. S7 corroborate the hypothesis that G1-type Gr remains in the growth stage when the deposition time is less than 2 h, and that the presence of G2-type Gr is absent in these conditions.

As clearly illustrated by the comparative spectra in Fig. S7b, the Raman spectra of Gr prepared at 1050 °C for varying deposition times are presented. Its structural characteristics are highly similar to those of Gr prepared at 1000 °C. When the deposition temperature exceeds 1050 °C, the temporal evolution of Gr structure characteristics, fully captured in Fig. S7c-e, manifests a distinct mode. It is evident from the spectral sequence that the nucleation of G2-type Gr has already begun to exist in 1100-1, 1150-1, and 1200-1. Consequently, the relative intensity of G1 peak gradually decreases, while the relative intensity of G2 peak gradually increases with the extension of deposition time. Furthermore, the I_D_/I_G_ increases with deposition time, which may be attributed to the ongoing nucleation of G2 type Gr, resulting in a substantial number of edge defects being contributed by small-sized carbon clusters. The calculated I_2D_/I_G_ ratio decreases with an increase in deposition time, indicating that the number of Gr layers continues to increase. However, the structural characteristics of Gr prepared at 1200 °C are slightly different from those at 1100 and 1150 °C (Fig. 4j, k). As supported in Fig. S7e, the I_D_/I_G_ decreases with an increase in deposition time and then tends to be stable. This phenomenon can be attributed to the growth of G2-type Gr nanosheets, concomitant with a decline in nucleation rate, leading to a reduction of edge defects. Figure S8 shows the cross‑section microstructure of 1200‑3, exhibiting a three‑layer configuration: a Pt protective top layer, an intermediate Gr layer, and an amorphous SiO_2_ fiber substrate at the bottom. Notably, the Gr layer displays a distinct bilayer morphology. The sub‑layer adjacent to the SiO_2_ substrate contains more edge defects, exhibits lower continuity, and has a thickness of about three layers, consistent with the G1‑type Gr identified by Raman analysis. In contrast, the upper sub‑layer grown above it shows fewer defects and improved continuity, corresponding to the G2‑type Gr characterized in the Raman results.

Based on the Raman shift difference between G1 and G2 peaks, G1 exhibits higher edge defect density and smaller crystallite size, whereas G2 features larger crystallites. This divergence primarily may originate from substrate effects. To further clarify the roles of CH_3_·, C_2_H_2_, and C_6_H_6_ in Gr nucleation and growth, the adsorption energy calculations for these three precursors on different substrate surfaces were supplemented. As shown in Fig. 4m, the adsorption energies of CH_3_·, C_2_H_2_, and C_6_H_6_ on the initial SiO_2_ substrate are − 1.001, − 1.016, and − 0.987 eV, respectively. However, on the SiO_2_ substrate covered by three layers of graphene (SiO_2_-Gr3, structure shown in Fig. 4l), their adsorption energies change significantly to − 0.053, − 0.098, and − 0.802 eV, respectively. The results indicate that CH_3_· acts as the primary driver for early stage nucleation due to its strong adsorption on SiO_2_. To clarify why 3-layer Gr mark the growth transition, we constructed five surface models: pure SiO_2_, and SiO_2_ covered with 1 to 4-layer Gr (SiO_2_-Gr1 to Gr4). The calculated CH_3_· adsorption energies are − 1.001, − 0.433, − 0.075, − 0.053, and − 0.050 eV, respectively (Fig. 4n). The adsorption energy increases with the Gr layer number, indicating progressively weaker adsorption. Notably, after three layers, the energy stabilizes, showing that three Gr layers sufficiently screen the SiO_2_ substrate effect. Hence, the disappearance of the substrate effect at three layers is the key transition point, shifting the growth mechanism from CH_3_· dominated rapid nucleation to C_6_ promoted grain growth. As illustrated in Fig. 4o–q, the diffusion energy barriers of three primary monomers on two distinct substrate surfaces are demonstrated. The diffusion energy barriers of CH_3_· on SiO_2_ and Gr substrates are 0.54 and 0.43, respectively. The diffusion energy barriers of C_2_H_2_ on SiO_2_ and Gr substrates are 1.77 and 1.55, respectively, while those of C_6_H_6_ are 0.77 and 0.49, respectively. The results demonstrate consistently higher diffusion barriers on SiO_2_ versus Gr for all precursor species. This indicates suppressed precursor mobility on SiO_2_, inhibiting large crystallite formation and promoting edge defect generation. These computational findings explained that the reason of G1-type Gr contains more defects than G2-type Gr.

Different radicals precisely regulate nucleation and growth through their specific adsorption and diffusion behaviors. C_1_ radicals (CH_3_·): High activity, strong adsorption and difficult diffusion on the initial SiO_2_ surface, inducing high-density random nucleation, resulting in small grain size and numerous grain boundary defects [19, 21]. C_2_ species (C_2_H_2_): The weak adsorption and diffusion capabilities of the Gr surface limit its ability to promote growth [36]. C_6_ species (C_6_H_6_): Possessing moderate adsorption energy and excellent diffusivity, capable of long-range migration and perfect epitaxial incorporation at lattice edges, effectively suppressing secondary nucleation, thereby dominating the growth of large-grained, low-defect, high-quality Gr [37]. Temperature-mediated tuning of the ratio among the three enables a switch in growth mode from “defect engineering” to “crystallization optimization”.

**Fig. 5:**
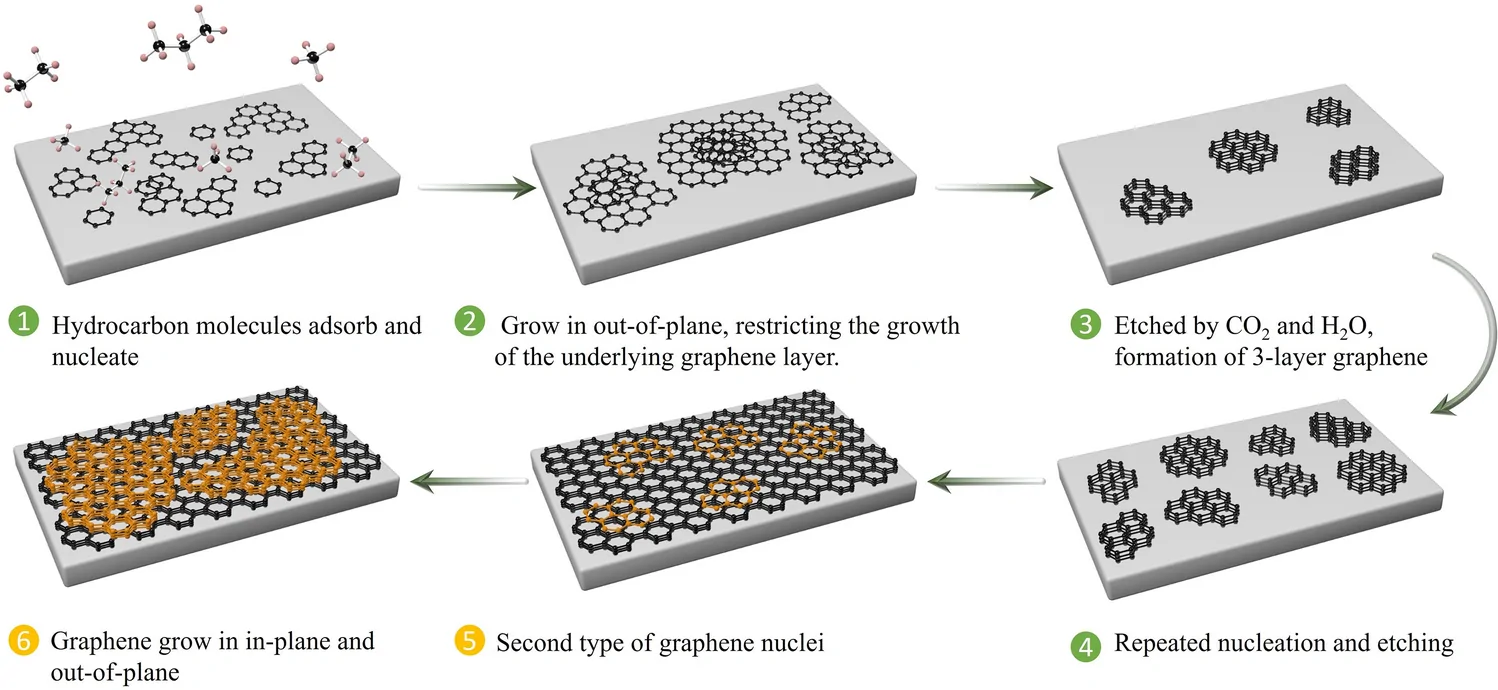
Mechanisms of Gr deposition using CH_3_OH as a carbon source

In the initial deposition stage (Fig. 5), the pyrolysis products are predominantly linear small molecules such as CH_3_· and C_2_H_2_, which govern the nucleation and growth of the G1-type Gr layer. These species readily adsorb onto the SiO_2_ substrate, promoting rapid nucleation and initiating growth. After the out-of-plane deposition of three Gr layers, the adsorption energy of CH_3_· decreases significantly, slowing further nucleation and growth. Concurrently, H_2_O and CO_2_ etch the edges, re-exposing active sites and accelerating lateral expansion. This stage is nucleation‑dominated, yielding small Gr grains with a high density of grain‑boundary defects. Once the G1-type Gr layer fully covers the substrate, the growth interface shifts to the Gr surface. At this stage, the adsorption of CH_3_· and C_2_H_2_ on Gr weakens considerably, reducing the nucleation rate. C_6_H_6_, with its moderate adsorption energy and high mobility, undergoes long‑range diffusion and preferentially attaches to low‑energy sites such as step edges, effectively suppressing random secondary nucleation. This stage is growth‑dominated, leading to larger Gr grains with fewer grain‑boundary defects.

### Electrical and Thermal Properties

Deposition temperature governs the types and concentrations of radical species in CH_3_OH pyrolysis, while differences in deposition times fully reflect the influence of substrate effects on carbon cluster migration. Through synergistic interplay of radical regulation and substrate effects, precise microstructure engineering of Gr becomes achievable.

**Fig. 6:**
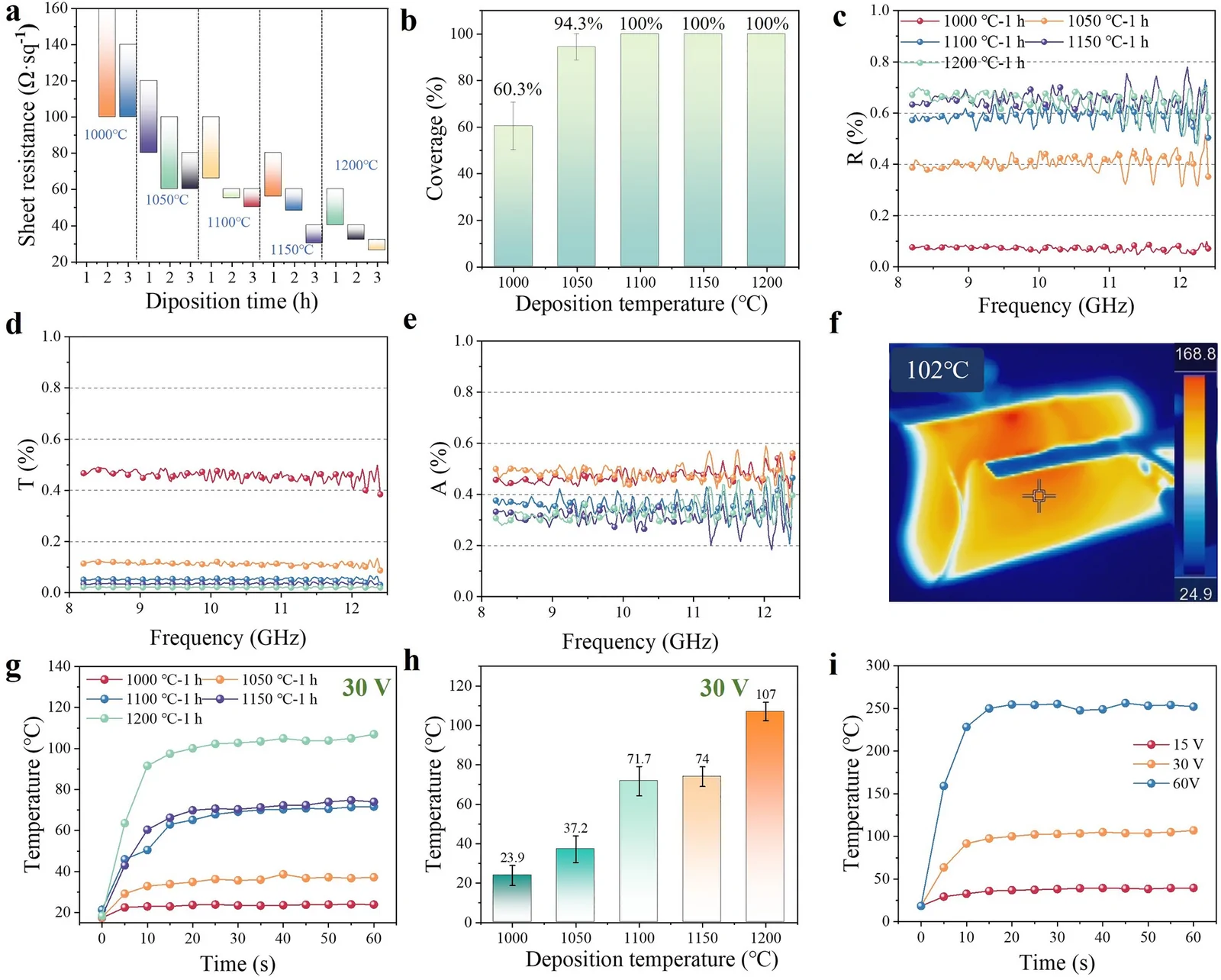
**a** Sheet resistance of Gr-skinned fabric under different deposition parameters. **b** Gr coverage at different deposition temperatures. **c** Reflectance, **d** transmittance, **e** absorptance of Gr fabric at different deposition temperatures. **f** Infrared image of Gr-skinned fabric during Joule heating, **g** temperature–time curve at an applied voltage of 30 V, **h** temperature uniformity on a Gr-skinned fabric at an applied voltage of 30 V, **i** temperature–time curve of 1200 °C-1 h under different voltages

The electrical properties of Gr are closely related to its microstructure, whereby a higher Gr content and texture result in enhanced electrical conductivity of the composites. Therefore, the sheet resistance (*R*_s_) of Gr-skinned fabric prepared under different deposition processes was tested (Fig. 6a). 1000-1 failed to form a continuous conductive pathway of Gr due to the slow deposition rate of Gr, thus resulting in open circuits. The lowest *R*_s_ of 1050-1, 1100-1, 1150-1, and 1200-1 are 80, 66, 56, and 40 Ω sq^−1^, respectively. It has been demonstrated that *R*_s_ decreases gradually with increasing deposition temperature. As shown in Fig. 6b, the coverage rates of samples 1000-1 and 1050-1 are 60.3% and 94.3%, respectively, while samples prepared at higher temperatures achieve 100% coverage. Notably, when the temperature exceeds 1050 °C, a marked decrease in *R*_s_ is observed, which can be attributed to the complete and uniform coverage of Gr on the substrate surface, thereby forming a continuous conductive pathway. Systematic measurements at 1000 °C over different deposition times reveal that the Rs decreases exponentially with increasing coverage, confirming that the failure to reach the percolation threshold is the primary cause of the high resistance observed in the early stage samples (Fig. S9). For 1200-3 with in-plane size of 180 × 180 mm, the lowest Rs is as low as 26 Ω sq^−1^, and the highest Rs is only 32 Ω sq^−1^, indicating that high deposition temperatures can achieve uniform deposition of large-size fabric under long deposition times. Existing strategies primarily focus on achieving wide *R*_s_ ranges (2-16 k, 50-2.8 k, 25-300, 11–150 Ω sq^−1^) often via extended deposition or post-growth modifications [22, 38–40]. In contrast, our radical-manipulation CVD process emphasizes precise, fine-grained control within a practically optimal mid-range (26–150 Ω sq^−1^). This is quantitatively superior, yielding the lowest average division value (defined as the total *R*_s_ tuning range divided by the number of discrete sample points within that range) of 8.8 Ω sq^−1^, which demonstrates a unique capability for fine electrical property tuning essential for advanced multifunctional design.

Electromagnetic parameters of Gr-skinned SiO_2_ fabric were characterized via waveguide methodology, measuring S_11_ and S_21_ to calculate reflectance (R), transmittance (T), and absorptivity (A) (Fig. 6c–e) [41, 42]. The EMW absorption in Gr originates from the synergistic effect of conductive loss and defect-induced dipole polarization loss. The overall performance is governed by the combined influence of electrical conductivity, defect density, and the resulting impedance matching. R progressively increases with deposition temperature, exceeding 60% above 1100 °C. T exhibits inverse correlation, undergoing rapid decline beyond 1000 °C. Absorptivity demonstrates a non-monotonic profile, showing strongest EMW attenuation in 1100-1. Samples with high conductivity (1150-1, 1200-1) exhibit strong conduction loss, but their severe impedance mismatch with free space leads to high R, thereby limiting A. Conversely, samples with low conductivity (1000-1, 1050-1) show good impedance matching but possess insufficient intrinsic loss capability. The unique sample 1100-1 achieves an optimal balance: Its moderate conductivity improves impedance matching, allowing more EMWs to penetrate, while its higher defect density provides significant dipole polarization loss. Consequently, this sample attains the highest absorption performance by optimizing the synergy among conduction loss, polarization loss, and impedance matching.

R, T, and A describe “how the energy is distributed” at the material interface, while shielding effectiveness (specifically, absorption shielding effectiveness here) describes “how much the energy is attenuated” as the EMW propagates through the material. These two parameters characterize the material’s electromagnetic interaction performance from different dimensions. Total shielding effectiveness (SE_T_) increases proportionally with temperature, comprising reflective (SE_R_) and absorptive (SE_A_) components representing the ratios of reflected-to-incident waves and absorbed-to-transmitted waves, respectively (Fig. S10) [43, 44]. It can be observed that the magnitude of SE_R_ and SE_A_ (Fig. S10b, c) is positively correlated with the electrical conductivity of the materials. This indicates that, disregarding the absolute value of the absorption coefficient (which is influenced by impedance matching), Gr-skinned fabrics with higher conductivity possess a stronger intrinsic capability to attenuate electromagnetic waves. This observation aligns with the generally accepted electromagnetic wave loss mechanism for conductive wave-absorbing materials, where conduction loss plays a dominant role. More importantly, dominant SE_A_ over SE_R_ reveals absorption-driven shielding, wherein incident waves are primarily dissipated through conductive pathways in Gr networks and dipole polarization at defect sites.

To evaluate the Joule heating performance of Gr-skinned fabrics, a simplified heater was fabricated. Copper foil electrodes were attached to fabric terminals and connected to a DC power supply, with temperature distribution monitored in real-time via infrared thermography. For 100 × 100 mm^2^ fabrics, infrared image shows the temperature distribution of the Gr-skinned fabric under bending at thermal equilibrium (Fig. 6f). Figure 6g presents temperature–time curves across deposition temperatures. 1000-1 and 1050-1 exhibited negligible heating at 30 V due to high *R*_s_. As shown in Fig. 6h, the Gr-skinned fabric exhibits a favorable uniform temperature distribution across its surface upon reaching thermal equilibrium. Elevated voltages increased heat generation following Joule law, raising radiation temperatures (Fig. 6i).

**Fig. 7:**
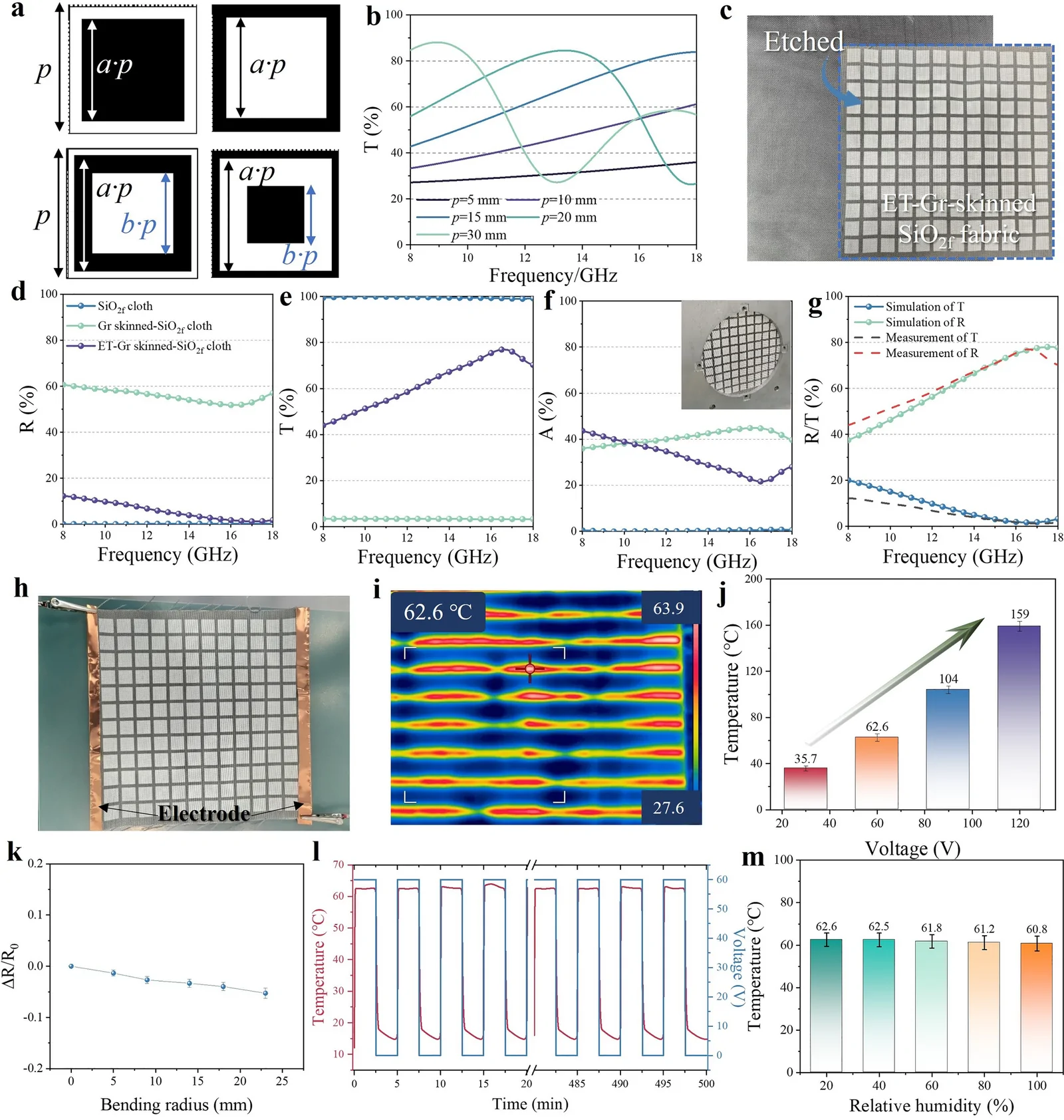
**a** Schematic diagram of traditional FSS configurations, **b** transmittance of band-pass type FSS under different structural parameters. **c** Digital images of ET-Gr-skinned fabric before and after etching. **d** Reflectance, **e** transmittance, **f** absorptance of the three types of fabric (SiO_2_ fabric, Gr-skinned fabric, and ET-Gr-skinned fabric), **g** simulated and measured transmittance.** h** Heating device for ET-Gr-skinned fabric, **i** infrared image of ET-Gr-skinned fabric, **j** temperature of ET-Gr-skinned fabric at different voltages, **k** electrical-resistance variation with bending radii from 5 to 23 mm, **l** temperature curves while applying a square wave from 0 to 60 V with period of 5 min for 100 cycles, **m** surface temperature at different relative humidity

It is evident that the currently developed Gr-skinned fabric cloth lacks efficient EMW transmission capabilities. To concurrently satisfy both communication and Joule heating requirements, implementation of meta-structure for EMW modulation is necessary. Frequency selective surface (FSS) achieves modulation of EMW reflection, absorption, and transmission through periodic patterning of continuous metallic or resistive surfaces. Conventional FSS configurations are categorizable into band-pass, band-stop, high-pass, and low-pass types, as illustrated in Fig. 7a. The Gr prepared at 1200 °C possesses a superior texture and lower electrical resistance, resulting in reduced ohmic loss under an electromagnetic field. Therefore, we selected the sample of 1200-1 to evaluate its EMW transparent and electrothermal performance. T of 1200-1 was calculated for these configurations (Figs. 7b and S11). Band-pass type structures exhibit low T at low frequencies and high T at high frequencies, whereas band-stop types demonstrate inverse characteristics. Crucially, resistive high-pass and low-pass configurations diverge significantly from their metallic counterparts due to ohmic losses, which eliminate abrupt cut-off behavior. Moreover, Gr must form a continuous in-plane conductive network to enable Joule heating functionality. Consequently, band-stop and low-pass configurations are unsuitable for this application. Reduced Gr coverage area predictably enhances T, but this simultaneously degrades Joule heating performance. Following comprehensive evaluation, the band-pass FSS configuration was selected for the integrated EMW communication and Joule heating Gr-skinned fabric. Structural parameter optimization yielded final dimensions of periodicity *p* of 15 mm and aperture ratio *a* of 0.76 [45, 46].

Based on the optimized FSS configuration, the Gr-skinned fabric was processed using laser scanning technology to create the meta-surface structured sample shown in Fig. 7c. The laser exerts both pronounced mechanical impact and thermal effects on Gr. By precisely controlling the laser power and repetition rate, selective and efficient etching of Gr‑skinned fibers can be achieved without damaging the substrate, as shown in Fig. S12. The etched fiber substrate remains intact, with no signs of fracture or melting. The R, T, and A of SiO_2_ fabric, Gr-skinned fabric, and etched (ET) Gr-skinned fabric were characterized via the free-space method (Fig. 7d–g). The SiO_2_ fabric exhibited low R and high T, which attributable to its low permittivity and minimal thickness. In contrast, the Gr-skinned fabric showed high R (50%-60%) and A (35%-45%), with T limited to 3.3% within 8–18 GHz. After etching, the ET-Gr-skinned fabric showed significantly reduced R and enhanced T, achieving a maximum T of 77%. Its transmission characteristics exhibited frequency-selective behavior: low transmission at lower frequencies and high transmission at higher frequencies. Defining the effective transmission band (ETB) as the frequency range where transmission (T) exceeds 60%, the ET-Gr-skinned fabric exhibits an ETB of 5.66 GHz (12.34–18 GHz). Figure 7g confirms strong agreement between simulated and measured R/T values for the ET-Gr-skinned fabric. Figure 7h illustrates the Joule heating test setup for the ET-Gr-skinned fabric. Under the voltage of 60 V, infrared thermography (Fig. 7i) revealed a striped temperature distribution with localized heating up to 62.6 °C. Voltage-dependent heating tests (Fig. 7j) demonstrated monotonic temperature increase with higher voltages. The ET-Gr-skinned fabric exhibits excellent stability for wearable applications. It shows remarkable mechanical robustness, with a relative resistance change (ΔR/R_0_) below 0.05 under bending radii from 5 to 23 mm (Fig. 7l), due to its continuous conductive network. The fabric also demonstrates exceptional cyclic stability, maintaining a surface temperature fluctuation within ± 1 °C over 100 heating/cooling cycles (Fig. 7l), owing to the high crystallinity of the CVD-grown Gr. Furthermore, its Joule heating performance shows near immunity to humidity. The intrinsically hydrophobic surface ensures stable resistance, with only a subtle temperature decrease above 40% relative humidity attributable to enhanced evaporative cooling (Fig. 7m).

**Fig. 8:**
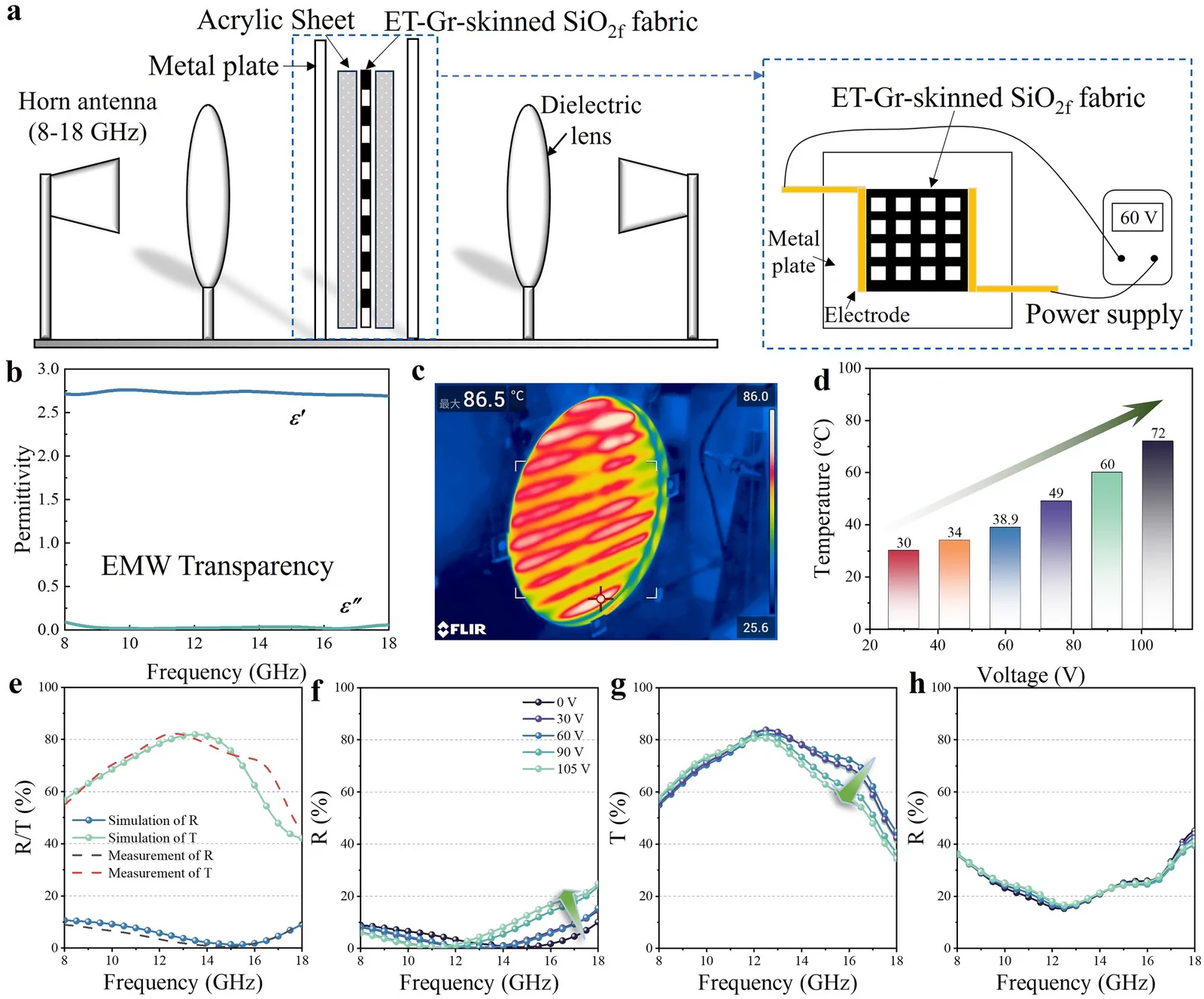
**a** Free-space synchronous Joule heating test system. **b** Permittivity of acrylic. **c** Infrared image of the sandwich structure during Joule heating, **d** surface temperature of the sandwich structure at different voltages. **e** Simulated and measured transmittance values of the sandwich structure, **f** reflectance, **g** transmittance, **h** absorptance at different applied voltages

Owing to the impracticality of standalone deployment for fabric, we fabricated a sandwich structure by laminating it with acrylic sheets. To better replicate real operational conditions, a Joule heating system was integrated into the free-space measurement setup (Fig. 8a), enabling simultaneous electromagnetic characterization during thermal activation. The acrylic serves as a wave-transparent material with a relative permittivity of 2.72 - 0.02 (Fig. 8b). Infrared thermography during free-space testing (Fig. 8c) reveals a striped temperature distribution across the heated surface, while voltage-dependent surface temperatures (Fig. 8d) indicate reduced heating efficiency compared to standalone ET-processed Gr-skinned fabric. Unheated electromagnetic simulations and measurements of the sandwich structure (Fig. 8e) demonstrate excellent agreement. The sandwich configuration of the ET-Gr-skinned fabric achieved a wider ETB of 8.48 GHz (8.62–17.1 GHz), resulting from the phase modulation of electromagnetic waves by the acrylic sheets. During Joule heating, electromagnetic parameter characterization showed that R and T spectra shifted toward lower frequencies with increasing voltage, whereas A exhibited negligible variation (Fig. 8f–h). Table 1 presents the maximum T, the corresponding ETB and surface temperature of the sandwich structure under different applied voltages.

**Table 1:** Transmittance and surface temperature of sandwich structure at different applied voltages

Voltage (V)	Surface temperature (°C)	Maximum transmittance		Effective transmission band (transmittance > 60%)		
		Position (GHz)	Value (%)	Position 1 (GHz)	Position 2 (GHz)	Value (GHz)
0	25	12.53	82.2	8.62	17.1	8.48
30	30	11.95	82.3	8.46	16.88	8.42
60	39	12.54	83.6	8.52	16.93	8.41
90	60	12.37	82.3	8.3	16.22	7.92
105	72	12.27	80.8	8.22	15.51	7.29

However, it must be acknowledged that the enhancement of Joule heating performance comes at the cost of a gradually narrowing ETB. As the Gr units constitute the sole loss component in this architecture, the minimal variation in A confirms the stable electrical resistance of Gr under heating at 30–120 V. To analyze the cause of the shift in the R and T curves during Joule heating, the complex permittivity of the acrylic material from 20 to 100 °C was measured, as shown in Fig. S13. The results indicate a gradual increase in the real part of the permittivity (*ε*′), while the imaginary part (*ε*″) shows relatively minor changes. Furthermore, we simulated the electromagnetic transmission characteristics of the sandwich structure with the acrylic layer at 80 °C and compared the results with those at room temperature (20 °C). The simulations reveal subtle differences in R and T at 80 °C (Fig. S14), primarily manifesting as a shift of the R, T, and A curves towards lower frequencies in the 14–17 GHz range. These simulation results are consistent with the measured electromagnetic transmission characteristics at applied voltages up to 105 V, indicating that the temperature-dependent dielectric properties of the acrylic are the main reason for the changes in transmission performance during Joule heating. The R and T spectral shifts primarily originate from the temperature-dependent permittivity of acrylic. As the temperature increases, the permittivity changes, thereby enhancing EMW reflection (particularly at higher frequencies). At an applied voltage of 105 V, the Joule heating effect raised the surface temperature of the sandwich structure to 72 °C. Simultaneously, the structure exhibited an ETB as wide as 7.29 GHz, covering most of the X and Ku bands, and a peak transmission of 80.8% at 12.27 GHz, demonstrating a collaborative balance between broadband EMW transmission and Joule heating. These collective results verify that the Gr-skinned fabric holds significant potential for applications in the electromagnetic-thermal field.

Metal-coated fibers offer the highest conductivity and heating efficiency, with *R*_s_ as low as 0.29 mΩ sq^−1^ [47–49]. However, they are highly reflective to EMW (near-zero transmittance) and are difficult to pattern non-destructively with lasers. Carbon nanotube (CNT) fabrics provide tunable conductivity (57–245 Ω sq^−1^) and mechanical flexibility, but their resistance typically remains in a higher range [50, 51]. Intrinsic conductive polymers like PEDOT:PSS allow some optical transmittance, yet exhibit low heating efficiency (*R*_s_ ~ 29 Ω sq^−1^ with multiple layers) and a narrow tunability range dependent on physical stacking [52]. In contrast, the methanol-CVD Gr-skinned fabric developed in this work achieves a finely tunable *R*_s_ (26–150 Ω sq^−1^), bridging the crucial low-to-medium range for multifunctional devices. It combines rapid thermal response, high-temperature stability, and excellent laser-pattern ability. Most notably, its patterned FSS structure enables significant EMW transmittance (up to 77%) while maintaining efficient Joule heating, a balanced synergy of tunability, multifunctionality, and stability not achievable with metal, CNT, or polymer-based counterparts.

Despite the demonstrated balance between EMW transmission and Joule heating, this work has limitations. The primary constraint stems from the non-uniform temperature distribution inherent to the FSS patterning, where heat generation is localized to the conductive grids. Future efforts to overcome this challenge should focus on enhancing the in-plane thermal transport of the system. This could be achieved by integrating highly thermally conductive, wave-transparent materials (BN or Si_3_N_4_) as either substrates or coatings, and by further optimizing the FSS geometry to improve heating uniformity. These strategies present a promising pathway toward advanced multifunctional fabrics with fully uniform thermal management and excellent electromagnetic compatibility.

## Conclusion

The methanol-CVD process enables precise microstructure control in Gr-skinned SiO_2_ fabric through radical manipulation and substrate engineering. Low temperatures favor C_1_ species, forming defective Gr, while high temperatures enrich C_2_/C_6_ aromatics, producing conductive Gr. The adsorption capacity of C_1_ varies significantly across different substrates. As the number of graphene layers on the SiO_2_ surface increases, the adsorption energy gradually rises, making it difficult for C_1_ to nucleate effectively. When the Gr layer count reaches three, the adsorption energy stabilizes, explaining the transition in SiO_2_ growth from a single layer to a bilayer structure. The unique bilayer Gr structure allows for precise tuning of its electrical resistance, with the *R*_s_ of the Gr-skinned fabric being adjustable within the range of 25–150 Ω sq^−1^. To validate its potential for integrated communication and electrothermal applications, a Gr-skinned fabric with an FSS configuration was fabricated. Band-pass FSS patterning via laser patterning enhances EMW transmittance from 3.3% to 77% while preserving Joule heating. A sandwich structure was prepared by laminating the Gr-skinned fabric with EMW transparent sheets, and the synergy between EMW transmission and Joule heating was validated. Sandwich structures maintain broadband transmission (> 60%) of 7.29 GHz and stable heating 72 °C at an applied voltage of 105 V. This work provides a scalable pathway for fabricating Gr-skinned fiber composites with electromagnetic-thermal functionality, overcoming conventional fiber limitations.

## Supplementary Information

Below is the link to the electronic supplementary material.

Supplementary file1 (DOCX 1704 KB)
