# Supplementary material for: Graphene-Skinned Fiber with Fine-Tunable Electrical Resistance via Radical and Substrate Engineering for Electromagnetic-Thermal Fabric
Source: Nanomicro Lett. 2026 Mar 2;18:263. doi: 10.1007/s40820-026-02117-8 (PMC12953830; doi:10.1007/s40820-026-02117-8)
Supplement: Supplementary file 1 — Supplementary file1 (DOCX 1704 KB) [file 40820_2026_2117_MOESM1_ESM.docx]

Supporting Information for

**Graphene-Skinned Fiber with Fine Tunable Electrical Resistance via Radical and Substrate Engineering for Electromagnetic-Thermal Fabric**

Jie Liang^1^, Zhaochen Li^1^, Fang Ye^1^*, Yuchen Cao^1^, Yi An^1^, Xiaomeng Fan^1^*, Qiang Song^1^*

^1^ Science and Technology on Thermostructural Composite Materials Laboratory, Northwestern Polytechnical University, Xi’an 710072, People’s Republic of China

*Corresponding authors. E-mail: [yefang511@nwpu.edu.cn](mailto:yefang511@nwpu.edu.cn) (Fang Ye); [fanxiaomeng@nwpu.edu.cn](mailto:fanxiaomeng@nwpu.edu.cn) (Xiaomeng Fan); [songqiang511@nwpu.edu.cn](mailto:songqiang511@nwpu.edu.cn) (Qiang Song)

**Supplementary Figures**


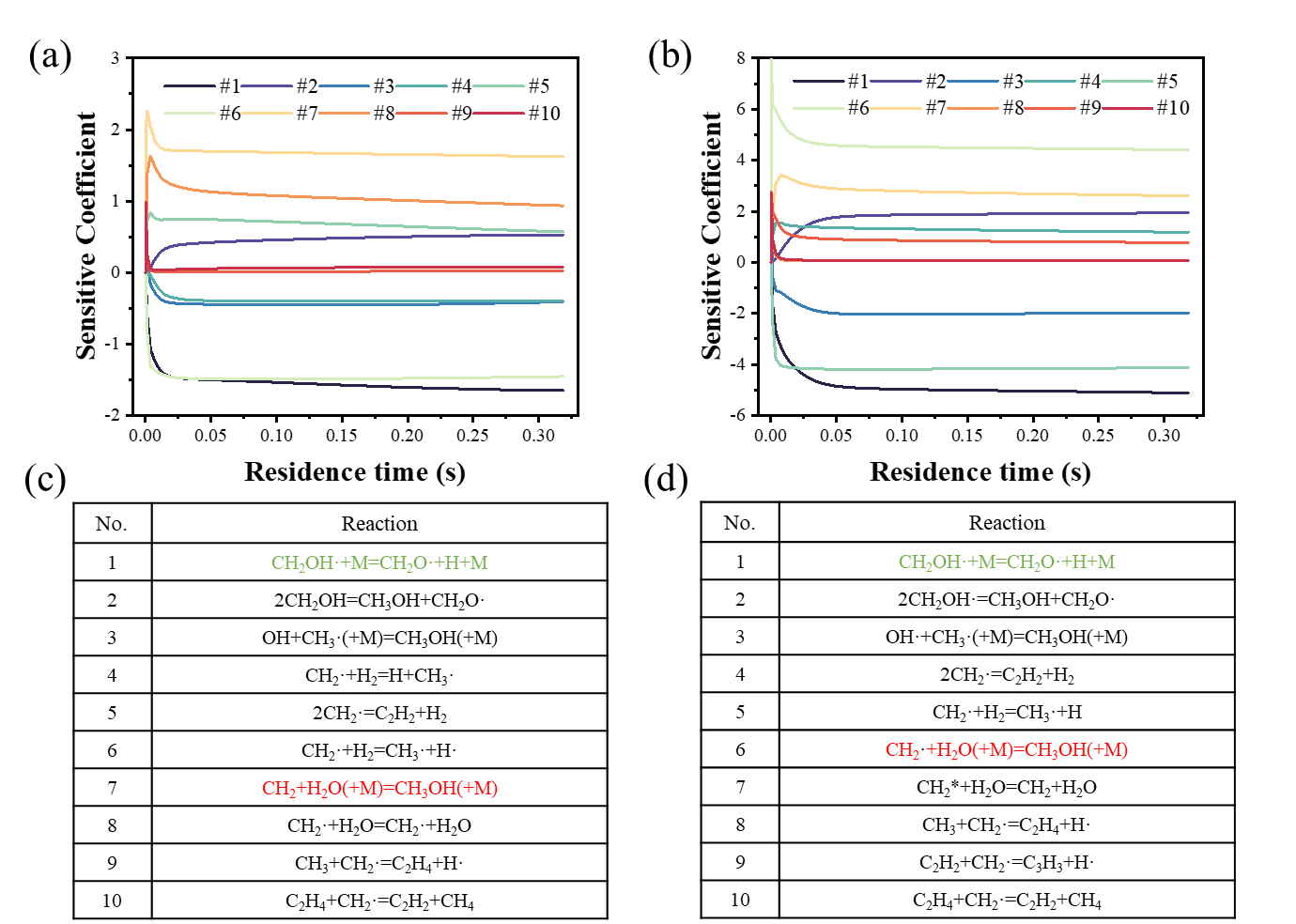


**Fig. S1** Sensitivity analysis of (**a**) C_2_H_2_, (**b**) C_6_H_6_, the top 10 elementary reactions affecting the sensitivity of the (**c**) C_2_H_2_, (**d**) C_6_H_6_


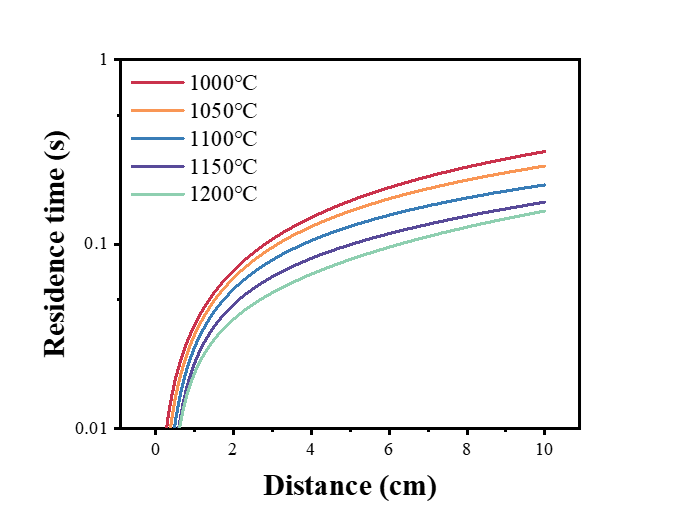


**Fig. S2** Residence time at different temperatures
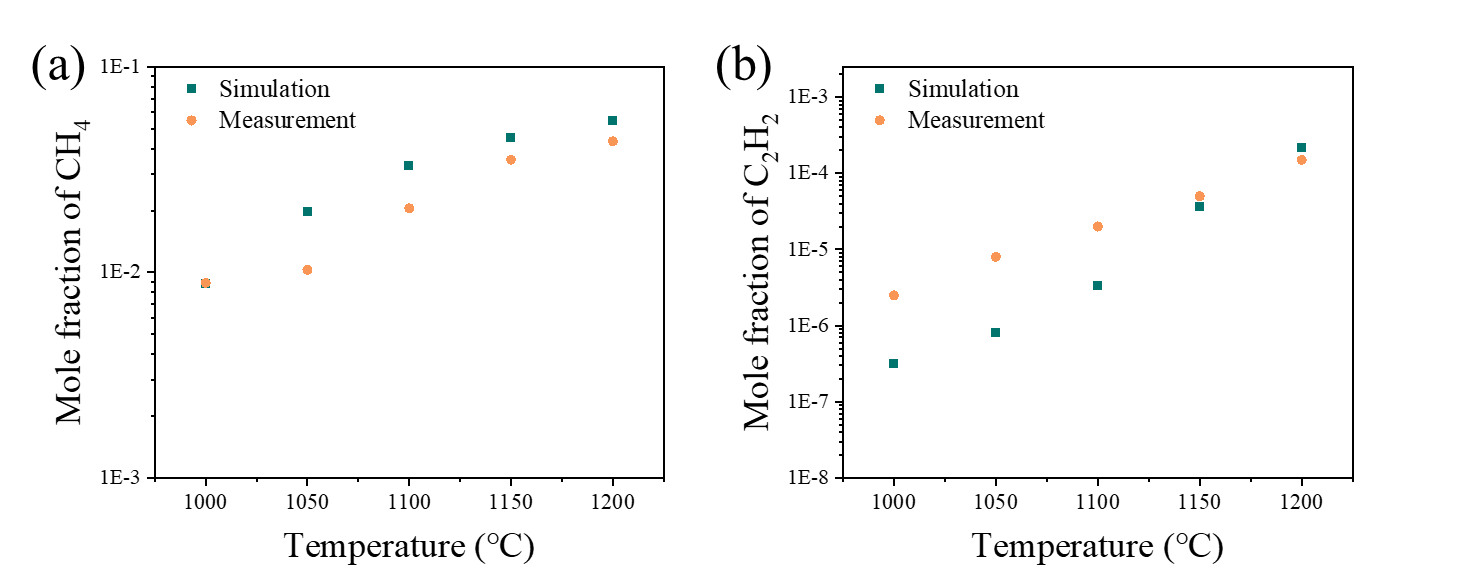


**Fig. S3** Variation of molar fraction of different gases with deposition temperature: (**a**) CH_4_, (**b**) C_2_H_2_


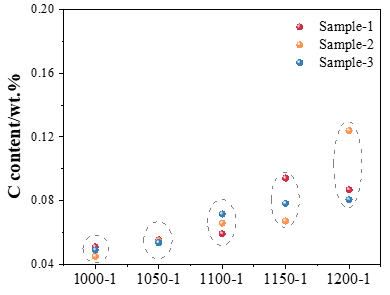


**Fig. S4** Carbon content in Graphene based SiO_2_ fiber cloth at different deposition temperatures


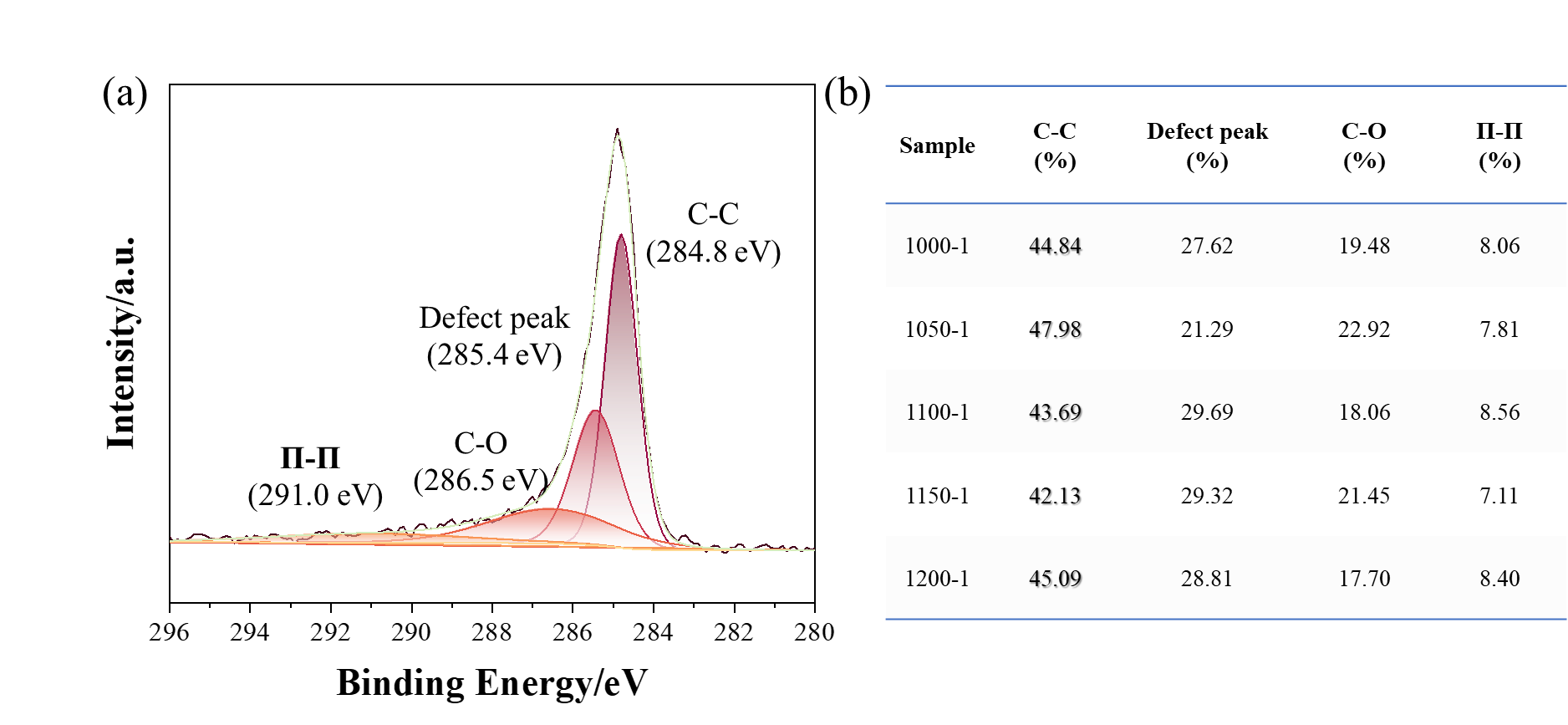


**Fig. S5** C 1s peak results of graphene at different deposition temperature: (**a**) spectrum, (**b**) bonding proportion


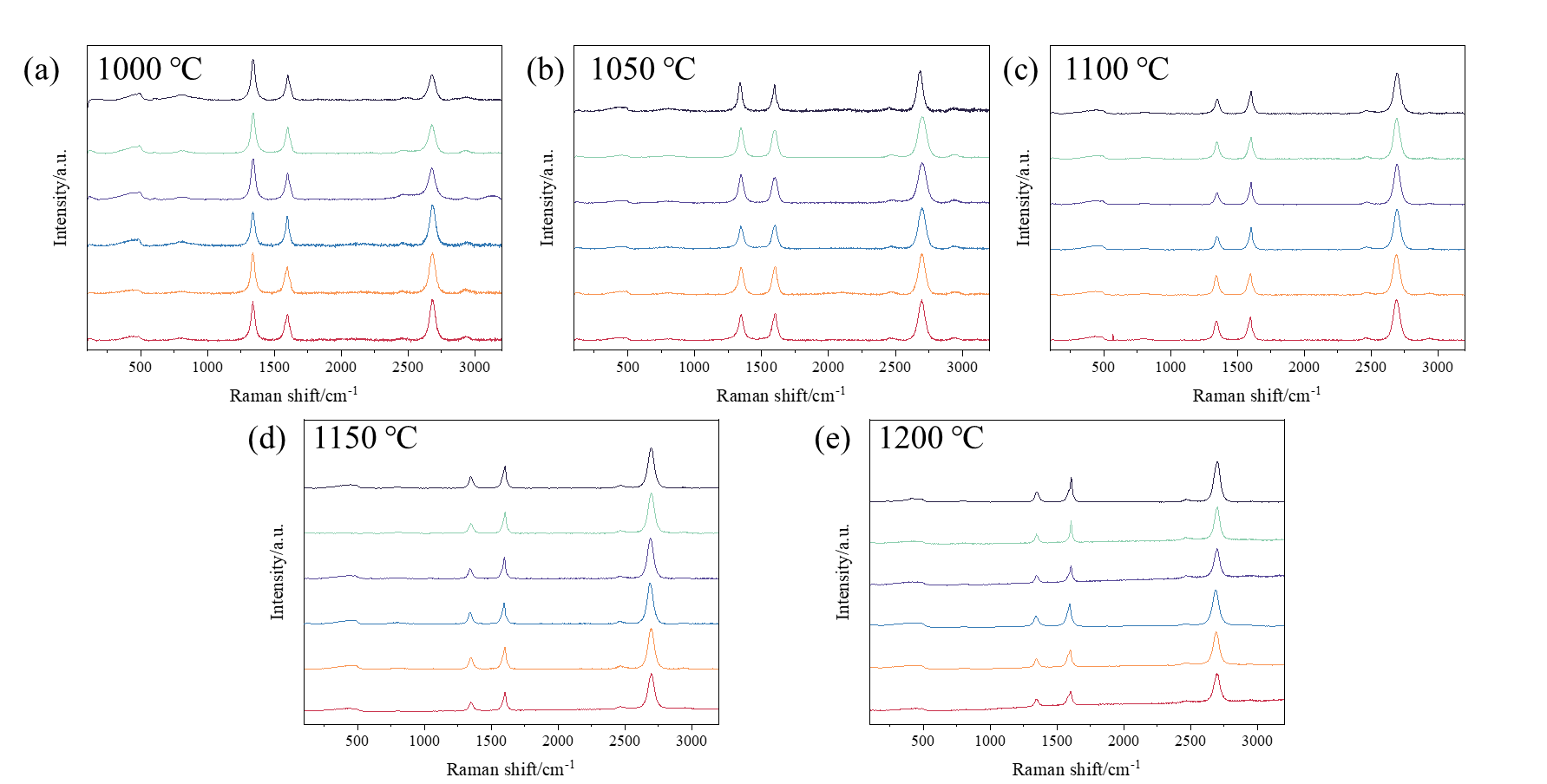


**Fig. S6** Raman spectra from different regions of the samples: (**a**) 1000-1, (**b**) 1050-1, (**c**) 1100-1, (**d**) 1150-1, (**e**) 1200-1


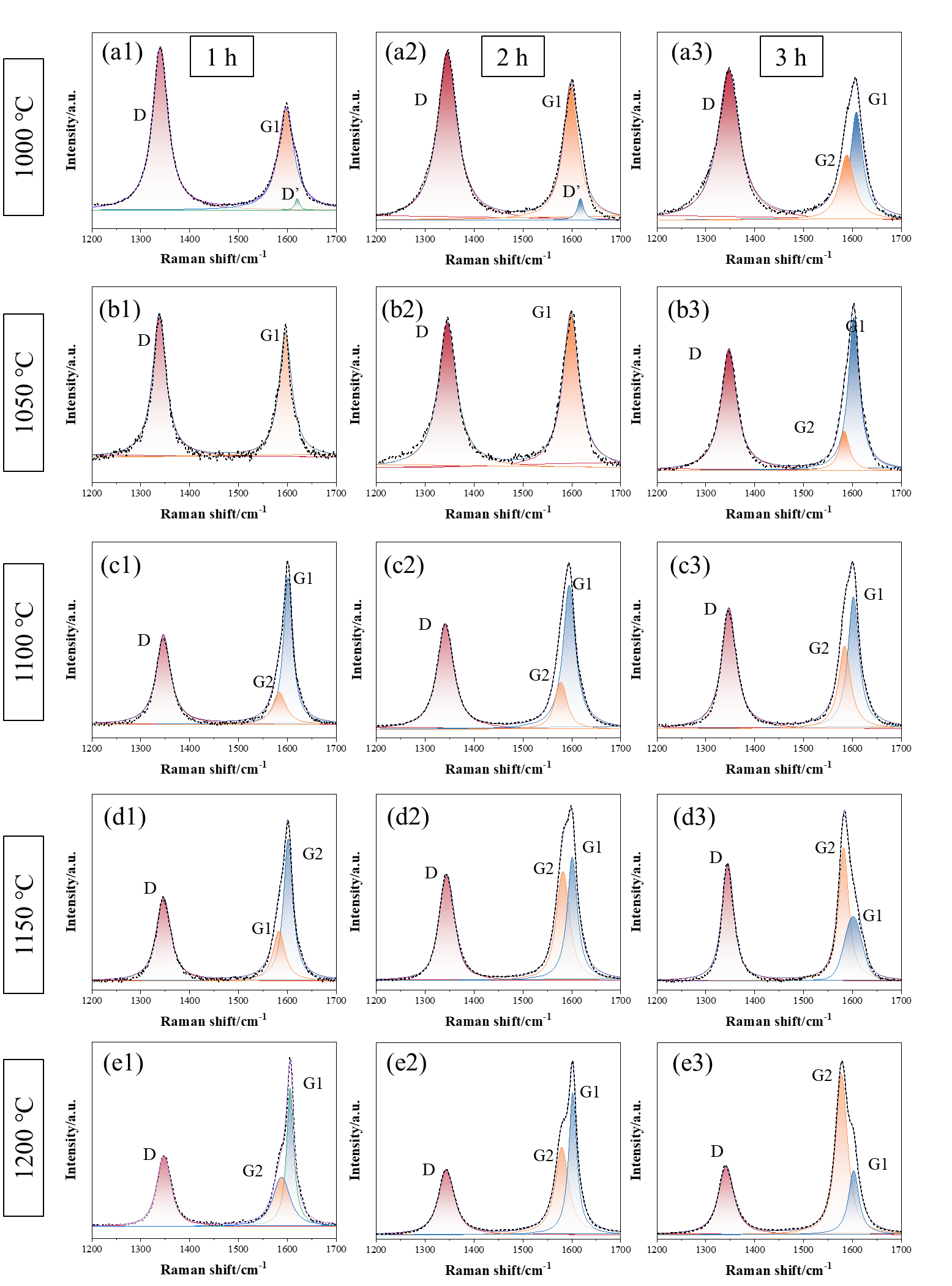


**Fig. S7** Raman spectra fitting results of graphene at different deposition times: (**a1**) 1000-1, (**a2**) 1000-2, (**a3**) T1000-3, (**b1**) 1050-1, (**b2**) 1050-2, (**b3**) 1050-3, (**c1**) 1100-1, (**c2**) 1100-2, (**c3**) 1100-3, (**d1**) 1150-1, (**d2**) 1150-2, (**d3**) 1150-3, (**e1**) 1200-1, (**e2**) 1200-2, (**e3**) 1200-3


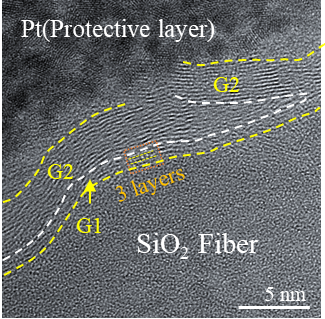


**Fig. S8** Cross-sectional microstructure of Gr-skinned SiO_2_ fiber


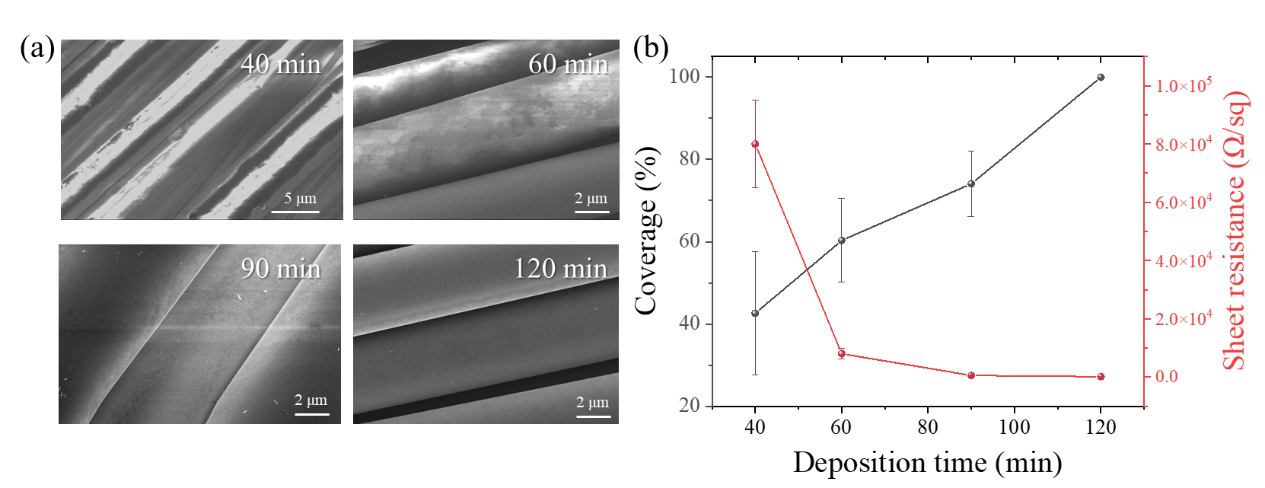


- **Fig. S9** (**a**) Micro-morphology and (**b**) coverage and sheet resistance at different deposition temperatures at 1000 ℃


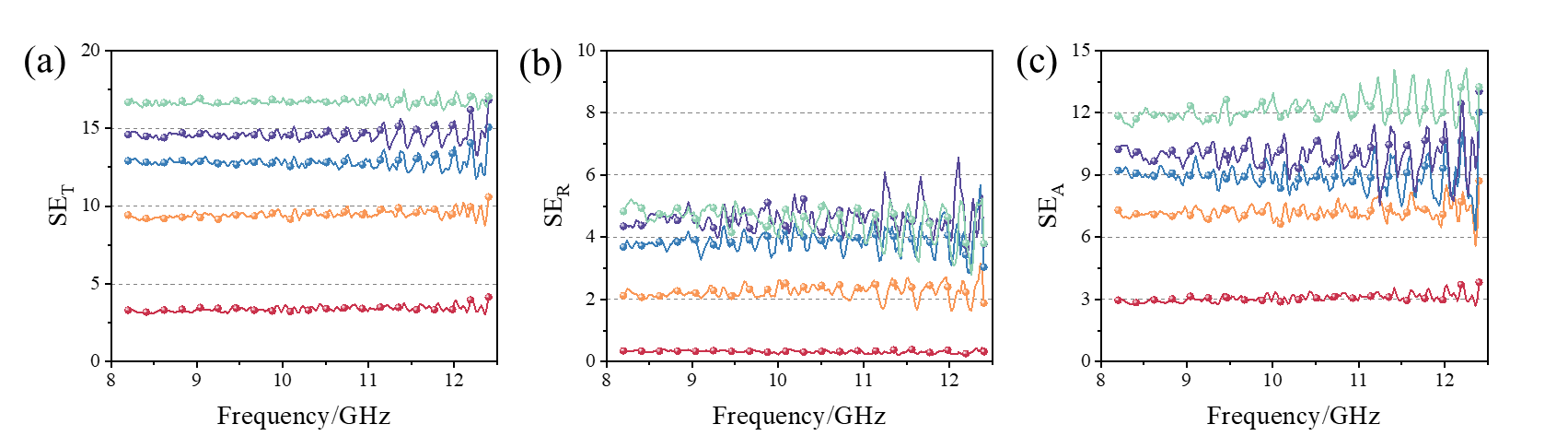


**Fig. S10** Electromagnetic wave response characteristics of graphene based SiO_2_ fiber cloth at different deposition temperatures: (**a**) total shielding effectiveness, (**b**) reflection shielding effectiveness, (**c**) absorption shielding effectiveness


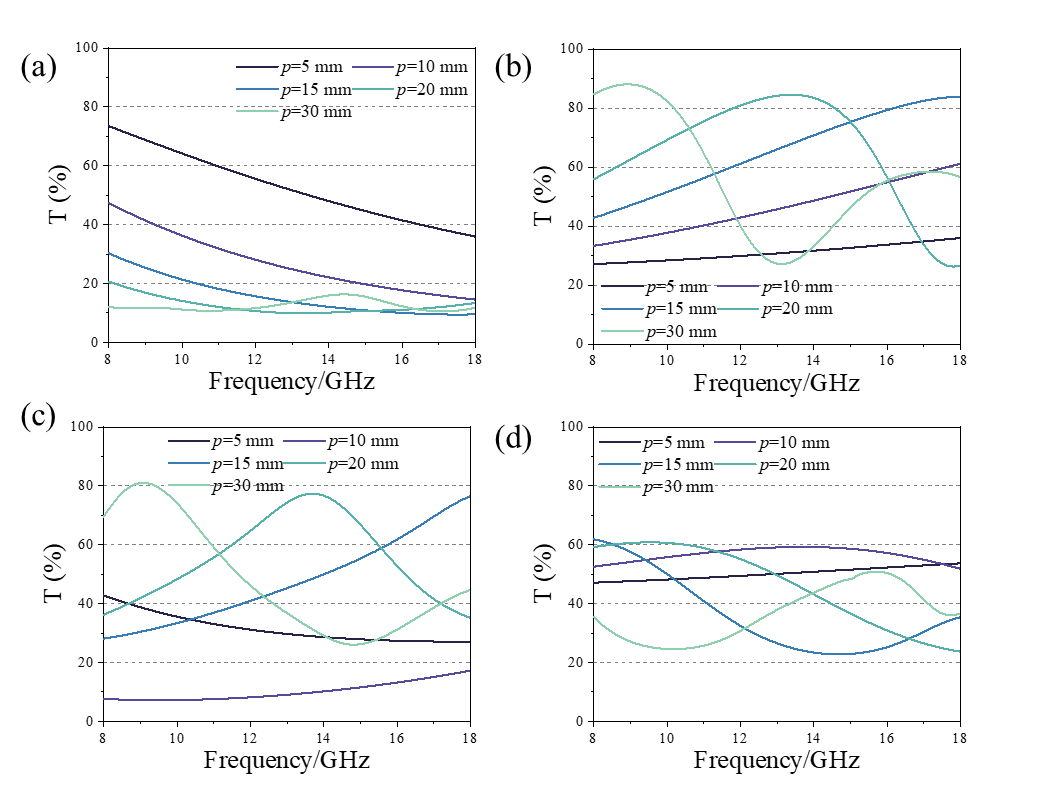


**Fig. S11** Transmittance under different structural parameters: (**a**) Band-stop type, (**b**) Band-pass type, (**c**) High-pass type, (**d**) Low-pass type


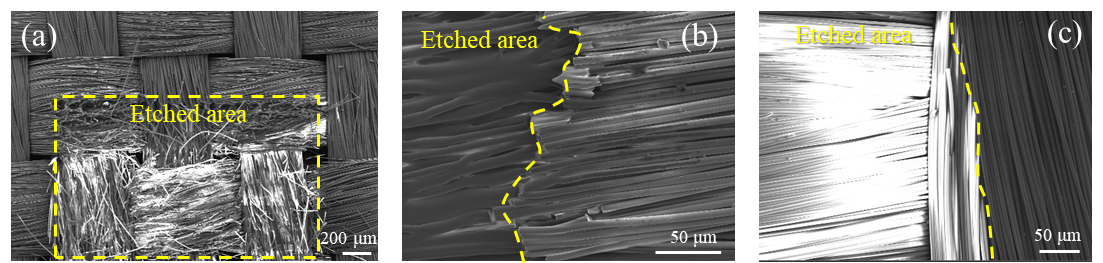


**Fig. S12** Etching phenomena under different laser parameters: (**a**) fiber damage, (**b**) fiber melting, (**c**) fiber remains intact


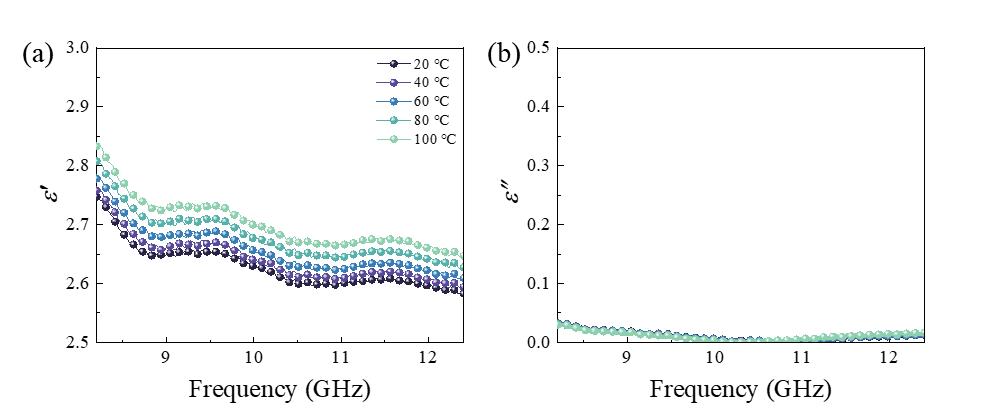


**Fig. S13** Dielectric constant of acrylic at different temperatures, (**a**) ε׳, (**b**) ε״


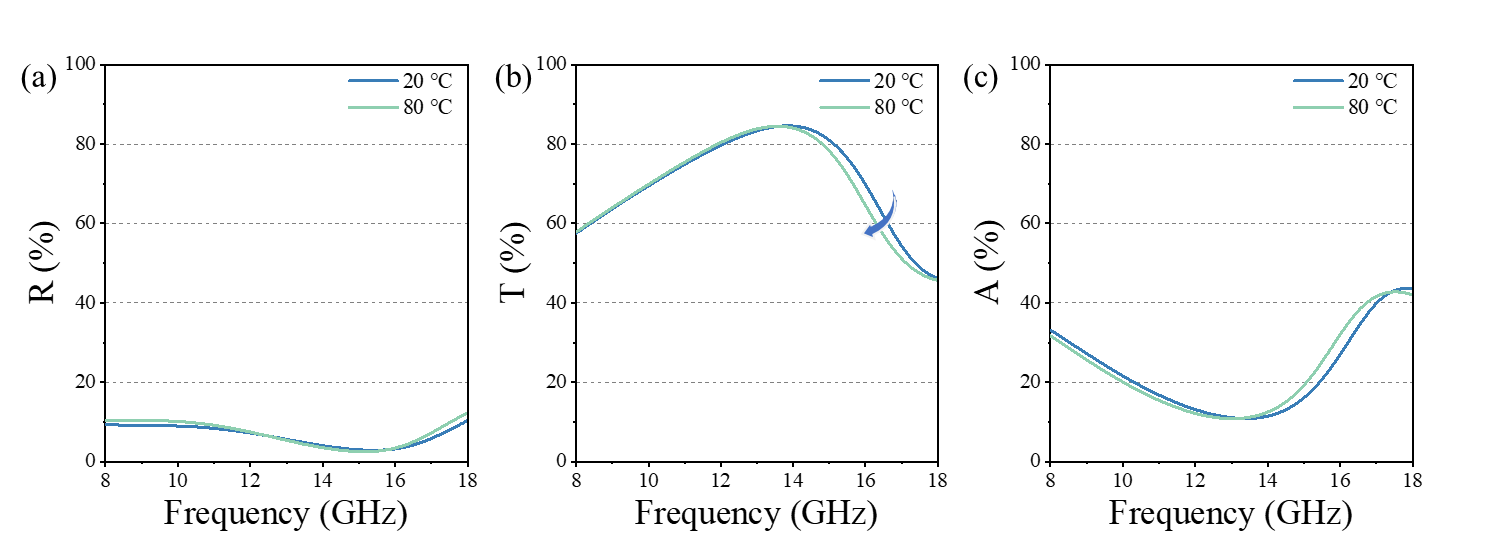


**Fig. S14** Simulated (**a**) Reflectance, (**b**) Transmittance, (**c**) Absorptance at 20 ℃ and 80 ℃
